# Effects of Tree Composition and Soil Depth on Structure and Functionality of Belowground Microbial Communities in Temperate European Forests

**DOI:** 10.3389/fmicb.2022.920618

**Published:** 2022-07-11

**Authors:** Luis Daniel Prada-Salcedo, Juan Pablo Prada-Salcedo, Anna Heintz-Buschart, François Buscot, Kezia Goldmann

**Affiliations:** ^1^Department Soil Ecology, Helmholtz-Centre for Environmental Research (UFZ), Halle, Germany; ^2^Department of Biology, University of Leipzig, Leipzig, Germany; ^3^German Centre for Integrative Biodiversity Research (IDiv), Leipzig, Germany; ^4^Department of Bioinformatics, Biocenter, University of Würzburg, Würzburg, Germany; ^5^Swammerdam Institute for Life Sciences, University of Amsterdam, Amsterdam, Netherlands

**Keywords:** bacterial pathways, deciduous/evergreen, fungal guilds, microbial indicator taxa, random forest, r/K-strategists

## Abstract

Depending on their tree species composition, forests recruit different soil microbial communities. Likewise, the vertical nutrient gradient along soil profiles impacts these communities and their activities. In forest soils, bacteria and fungi commonly compete, coexist, and interact, which is challenging for understanding the complex mechanisms behind microbial structuring. Using amplicon sequencing, we analyzed bacterial and fungal diversity in relation to forest composition and soil depth. Moreover, employing random forest models, we identified microbial indicator taxa of forest plots composed of either deciduous or evergreen trees, or their mixtures, as well as of three soil depths. We expected that forest composition and soil depth affect bacterial and fungal diversity and community structure differently. Indeed, relative abundances of microbial communities changed more across soil depths than in relation to forest composition. The microbial Shannon diversity was particularly affected by soil depth and by the proportion of evergreen trees. Our results also reflected that bacterial communities are primarily shaped by soil depth, while fungi were influenced by forest tree species composition. An increasing proportion of evergreen trees did not provoke differences in main bacterial metabolic functions, e.g., carbon fixation, degradation, or photosynthesis. However, significant responses related to specialized bacterial metabolisms were detected. Saprotrophic, arbuscular mycorrhizal, and plant pathogenic fungi were related to the proportion of evergreen trees, particularly in topsoil. Prominent microbial indicator taxa in the deciduous forests were characterized to be r-strategists, whereas K-strategists dominated evergreen plots. Considering simultaneously forest composition and soil depth to unravel differences in microbial communities, metabolic pathways and functional guilds have the potential to enlighten mechanisms that maintain forest soil functionality and provide resistance against disturbances.

## Introduction

Forest soil ecosystems hold high amounts of microbial biomass and diversity (Llado et al., 2018; He et al., 2020). Linked to the litter and exudate inputs from trees and understory vegetation, soil microbes sustain multiple ecosystem functions like decomposition, nutrient cycling, primary production, and multi-trophic interactions (Binkley and Giardina, 1998; Mori et al., 2017).

Soil microbial communities can be strongly affected by forest composition and are therefore known to react sensitively to forest conversion (Goldmann et al., 2015; Dukunde et al., 2019). Impacts of forest composition on soil microbes can be coupled to individual tree taxonomy and to differences at the functional level between evergreen and deciduous trees (Prescott and Grayston, 2013). Replacement of beech by spruce, for instance, is associated with changes in soil structure, including humus form and soil acidity, consequently impacting soil bacterial and fungal community composition (Berger and Berger, 2012; Nacke et al., 2016). Furthermore, divergent litter and root exudates of evergreen and deciduous trees can directly affect soil microbes (Eisenhauer et al., 2017). Indirectly, microbial communities are also shaped by interactions with other members of the soil food web (e.g., protists or nematodes), which in turn are also affected by forest tree composition (Bonkowski, 2004; Geisen et al., 2018). Litter fall often leads to an enriched microbial diversity and activity in the topsoil (Thoms et al., 2010; Uri et al., 2012; Ana et al., 2015). Consequently, and due to reduced vertical nutrient availability, it is assumed that bacterial and fungal diversity declines with increasing soil depth (Jobbágy and Jackson, 2001; Goebes et al., 2019). However, rhizodeposition affects microbes at deeper soil horizons (Certini et al., 2004; Lopez et al., 2020). Hence, microbial communities and their activities are differently affected by multiple factors in the topsoil compared to the subsoil (Blume et al., 2002; Loeppmann et al., 2016). However, frequently, only the top 10 cm of soil is studied, although microbial depth-dependence was recently reported: For instance, multiple bacterial and archaeal taxa in a poplar plantation exhibited significant changes across different soil depths, and indicated that main community transitions occurred at 10–20 cm (Feng et al., 2019). For soil fungi, a study in a *Betula albosinensis* forest detected that the abundance of the genus *Paxillus* decreased, whereas the abundances of *Inocybe* and *Genabea* increased with increasing soil depth (Du et al., 2017). Thus, ecological studies should consider at least the upper 30 cm (Richter and Markewitz, 1995; Goebes et al., 2019; Yost and Hartemink, 2020). While forest composition and soil depth are often considered independently, their interplay affects soil microbial communities, as for example, deciduous, evergreen, and mixed forests are related to different humus forms (Swift et al., 1979; Ponge, 2003). Likewise, soil profiles differ between deciduous and evergreen forests, where the former is characterized by strong mixing of organic material with the mineral soil components, and the latter by a distinct boundary between the mineral and organic horizons (Adams et al., 2019).

Although soil bacteria and fungi are affected by similar factors, and are closely interconnected, they differ in community structure, size, species richness, life history, and enzymatic capacity (Deveau et al., 2018). Therefore, bacterial and fungal assembly in relation to forest composition and soil depth varies from each other (Sun et al., 2017). In general, bacterial or fungal dominance depends on substrate degradation potential and nutrient availability (Van Der Heijden et al., 2008; Rinnan and Bååth, 2009). Fungi dominate soil systems with a high ratio of carbon to nitrogen (C:N), e.g., forest or no-till agricultural soils, while bacteria prefer systems with low C:N ratios, e.g., grasslands or conventional till agricultural soils (Moore et al., 2002). Consequently, different habitats shape specific microbial communities (Uroz et al., 2012; Bardgett et al., 2014). The divergence in environmental and nutritional requirements leads to niche differentiation and selection of organisms with different lifestyle strategies (Deveau et al., 2018).

Recent studies focusing on microbial functions, i.e., considering different bacterial metabolic pathways and fungal guilds, considered either the impact of forest composition or different soil depths. For instance, the use of predicted functional gene profiles based on 16S rRNA gene sequences facilitated the understanding of the vertical distribution of functional microbial groups (Uroz et al., 2013; Mushinski et al., 2018; Zhang et al., 2019) in relation to nutrient use across soil profiles in different ecosystems (Tripathi et al., 2019; Luan et al., 2020). Concerning fungal guilds, mycorrhizal fungal composition differed significantly in spruce compared to beech-dominated plots, and spruce-dominated plots had a higher abundance of saprotrophic relative to mycorrhizal fungi (Asplund et al., 2019). Likewise, analyzing fungal guilds in different soil layers indicated that competition for resources between saprotrophic and ectomycorrhizal (EcM) fungi led to restrictions in saprotrophic fungal dominance only in the topsoil in forests dominated by EcM trees (Carteron et al., 2020).

Considering the mentioned constraints, our study investigated how bacterial and fungal soil communities vary in relation to forest composition and three soil depths, while deciphering the potential microbial functional roles and contribution to forest ecosystem processes. We aimed to identify soil microbial taxa associated with deciduous forests, evergreen forests, or their mixtures vertically along the soil profiles until 30 cm depth. We expected that forest composition and soil depth exert different effects in shaping soil bacterial and fungal diversity and community structure. Precisely that microbial diversity decreases with deeper soil depth and an increasing proportion of evergreen trees. Due to their differences in the ability to process organic substrates, connection to plant roots, and cellular structures, we further anticipated that bacterial communities vary more than fungal communities along the soil profiles. In addition, we hypothesize that fungal communities are stronger shaped by forest composition than soil bacteria. At the functional level, we expect that bacterial pathway and fungal guild abundances will reduce with the deeper soil depth and with a high proportion of evergreen trees.

## Materials and Methods

### Study Sites

The study sites were composed of different “forest types” located in different European countries, which included boreal forests (Finland), hemi-boreal forests (Poland), and mountainous beech forests (Romania), all studied in the frame of the project SoilForEurope (http://websie.cefe.cnrs.fr/soilforeurope/). The plot design and sampling procedure have been described previously (Prada-Salcedo et al., 2021b). Our study used samples retrieved from 44 mature forest plots of 30 × 30 m^2^ from the FunDivEUROPE platform (Baeten et al., 2013). In spring 2017, the soil was sampled at subplot level (10 × 10 m^2^), considering five repetitions per plot using a split-tube sampler (diameter 5.3 cm). In addition, three different “soil depths” (0–10, 10–20, and 20–30 cm) were sampled from intact soil cores. Our plots contained monospecific and multispecies forest systems, and the “forest composition” categories consisted of either deciduous forests on eleven plots that comprise the tree species *Acer pseudoplatanus, Betula pendula, Carpinus betulus, Fagus sylvatica*, and *Quercus robur;* or evergreen forests on eleven plots including the tree species *Abies alba, Picea abies*, and *Pinus sylvestris*; or 22 mixed forests plots with evergreen and deciduous composition comprising eleven tree combinations (Supplementary Table 1).

### Molecular Procedures and Bioinformatics

Soil samples for molecular analyses were transported at 4°C and stored at −20°C until processing. The molecular procedure was described previously (Prada-Salcedo et al., 2021a,b). Briefly, total genomic DNA was extracted for each subplot and layer sample using the Power Soil™ DNA Isolation Kit (QIAGEN Laboratories Inc., Solana Beach, USA). After pooling DNA at the plot level, bacterial and fungal amplicon libraries were prepared by performing amplification of 16S rRNA gene V4 region (primers: P5_8N_515F and P5_7N_515F together with P7_2N_806R and P7_1N_806R) (Caporaso et al., 2011; Moll et al., 2018) and ITS2 (primers: P5-5 N-ITS4 and P5-6 N-ITS4 together with P7-3 N-fITS7 and P7-4 N-fITS7) (Gardes and Bruns, 1993; Ihrmark et al., 2012; Leonhardt et al., 2019), respectively. We performed a paired-end sequencing of 2 × 300 bp, using the MiSeq Reagent kit v3 on an Illumina MiSeq platform at the Department Soil Ecology of the Helmholtz-Center for Environmental Research—UFZ in Halle (Saale), Germany.

Raw sequences were extracted based on the presence of primers and then subsampled to 140,000 reads to compare samples in equal conditions. We used the standardized workflow of the dadasnake pipeline (Weißbecker et al., 2020; https://github.com/a-h-b/dadasnake), which is based on the DADA2 algorithm (Callahan et al., 2016). DADA2 produces amplicon sequence variants (ASVs), which bypass the previous common clustering at 97% sequence similarity. The ASV approach is more robust and realistic, and leads to a better resolution of microbial communities, especially in very rich environments such as soil (Joos et al., 2020). We used mostly default conditions of dadasnake pipeline, adjusting only the following parameters: minimum read length was set to 170 bp for bacterial read sequences and to 70 bp for fungal forward and reverse read sequences; maximum expected error after truncation was 0.5 for bacterial and 3 for each fungal sequence, respectively. Forward and reverse sequence variants were merged, and the chimeric sequences were removed by the consensus method. The bacterial ASVs were classified against the bacterial sequences in the SILVA database (Quast et al., 2013) (release 138). The prediction of functional profiles based on the bacterial ASVs was done using the Tax4Fun2 default database and the “group pathways” output (Wemheuer et al., 2020). Tax4Fun2 predicts gene abundances from 16S rRNA gene sequences and returns gene groups from the KEGG database that correspond to pathways at different levels, e.g., pathway IDs corresponding to Level 1: ko00190, ko00195, ko00680, ko00710, ko00720, ko00910, and ko00920; this ID pathways are corresponding to Level 2: oxidative phosphorylation, photosynthesis, methane metabolism, carbon fixation, photosynthetic organisms, nitrogen metabolism, and sulfur metabolism; and these pathways are grouped to the corresponding level 3: energy metabolism (see Supplementary Table 2 for all retrieved and used pathways). Fungal ASVs were checked and trimmed using ITSx (Bengtsson-Palme et al., 2013), and taxonomy was assigned using the UNITE (2017—Version 01.12.2017) database (Nilsson et al., 2019). Finally, we classified fungal guilds according to the assigned ASV taxonomy using FunGuild, using the main guilds of pathogens/parasites, saprotrophic, symbiotic (i.e., endophyte and epiphyte), mycorrhizal (i.e., ectomycorrhizal, arbuscular mycorrhizal, and orchid mycorrhizal), multi-lifestyle (taxa that shift guilds during their lifecycle), and undefined taxa (Nguyen N. H. et al., 2016).

### Statistical Methods

#### Abundances, Microbial Diversity, Microbial Community Composition, and Functional Abundances

From a total of 16,358 bacterial ASVs and 4,635 fungal ASVs, we evaluated the fungal and bacterial communities by using the R packages “phyloseq” (McMurdie and Holmes, 2013) and “vegan” (Oksanen et al., 2017) in the R software (R 2021-R version 4.1.2). For evaluation of abundances and microbial diversity, sequencing data was rarefied at the cutoff of 25,000 reads per sample for bacteria and 8,000 reads per sample for fungal communities, maintaining sample saturation and the balance between different soil depths (Supplementary Figure 1). Kruskal–Wallis tests followed by Dunn post-hoc tests were used to compare relative abundances at phyla level between forest composition and soil depth variables (Mendiburu, 2010). To evaluate relationships between microbial diversity, forest composition, soil depth, and possible interactions, we performed linear mixed models (LMMs) using forest type (country) as a random effect, since we consider this as the main source of variation, with the glmmTMB package (Brooks et al., 2017). Distances of microbial communities between different samples were calculated using Bray-Curtis dissimilarity, visualized by non-metric multidimensional scaling (NMDS), and statistically tested by permutational multivariate analysis of variance (PERMANOVA) using “vegan.” To see how forest composition and soil depth impacted microbial diversity at the functionality level, we used bacterial pathways and fungal guild abundances. The abundances of bacterial pathways were normalized by the “normalize” function with the “scale” method using the “BBmisc” package (Bischl et al., 2017). Then, the abundances of bacterial pathways and abundances of fungal guild were evaluated in relation to the proportion of evergreen trees in the plot and soil depth to fit generalized linear models (GLMs) with the glm function.

#### Random Forest Model Classifier for Forest Composition and Soil Depth Analysis

Random forest (RF) classifiers (Breiman, 2001) were trained on bacterial and fungal Hellinger-transformed abundance tables (Legendre and Gallagher, 2001), which could predict different classes (referred to as “labels”) of forest composition and soil depths from various microbial features (phyla, families, genera, and ASVs). In addition to the taxonomy levels, the Tax4Fun2-predicted functional profiles were used as features of specific bacterial pathways. For each feature-class pair, 100 random forests of 500 decision trees were trained using the default settings of the “randomForest” function implemented in the randomForest package (Liaw and Wiener, 2002), performed in the R and using 130 samples for bacteria and 131 samples for fungi. We kept the best random forest out of the 100 computed ones according to the out of bag (OOB) error. Furthermore, a group of 100 random forest classifiers was trained and tested by randomly created groups of 102 training samples and 29 testing samples for each feature-class pair. The test accuracy of each feature-class pair was averaged to validate the accuracy of the random forest models.

We obtained feature importance measures from the best RF according to the OOB error. These measurements were the mean decrease of the Gini index and the mean decrease in accuracy. The mean decrease of the Gini index gives an insight into how often a certain feature was successfully used to split the samples with respect to the used labels. Thereby, this importance measure is a proxy for an association which can be understood as an ecological indicator. Likewise, the mean decrease accuracy is obtained by permuting the value of the feature and evaluating the effect (decrease in accuracy) of the permutation (Breiman, 2001; Thompson et al., 2019). Neither importance measure necessarily indicates ecological importance, but signifies suitability as an indicator for a condition.

## Results

### Bacterial and Fungal Abundances, Diversity, and Community Composition in Relation to Forest Composition and Soil Depth

**Figure 1:**
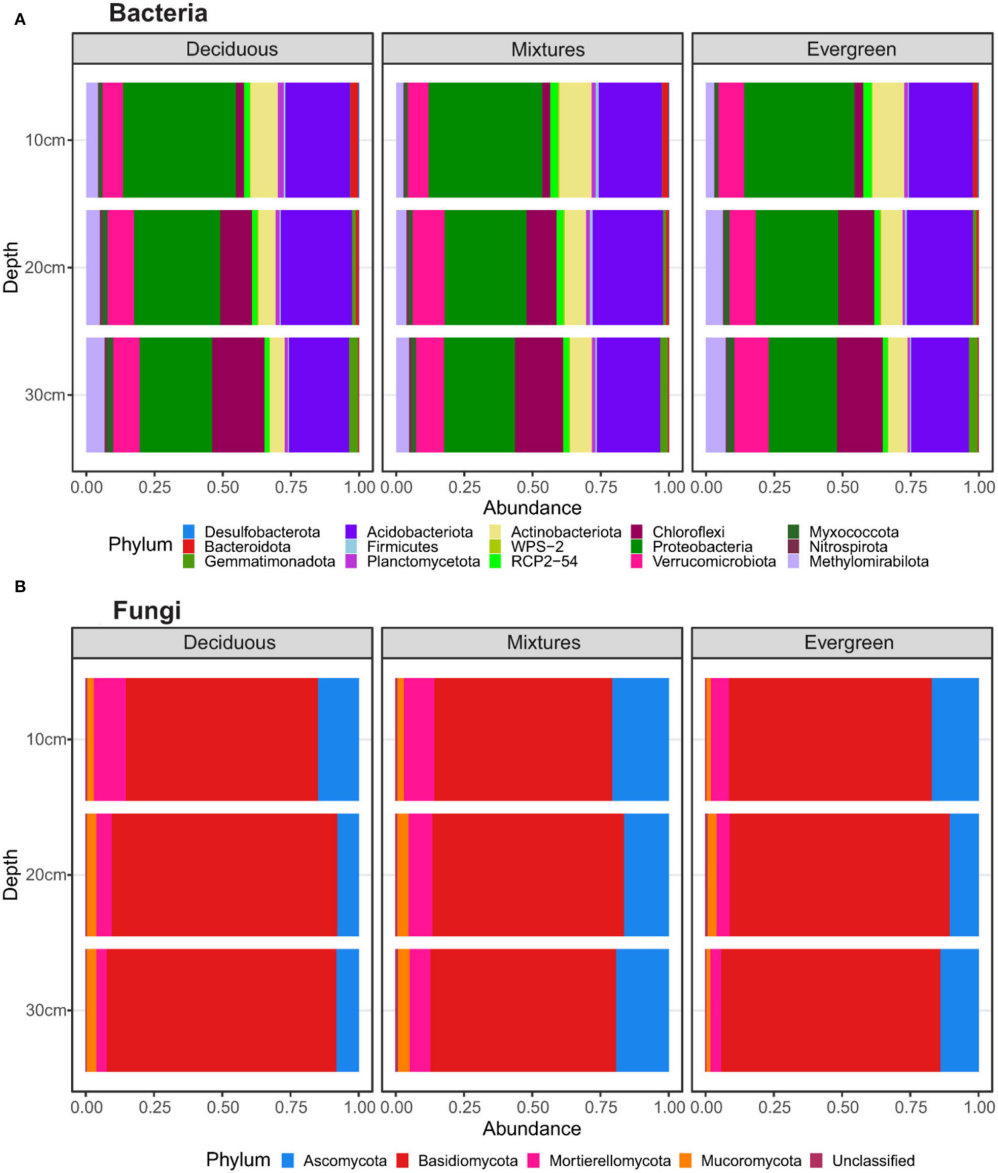
Relative abundances of **(A)** bacterial and **(B)** fungal phyla in relation to forest composition, split between deciduous, mixture, and evergreen forests (vertical panels), and soil depths. The data represent values of taxa with a relative abundance higher than 0.1 (for statistical details see Supplementary Tables 3, 4).

To gain a general overview of how forest composition and soil depth impact the community structure of soil bacterial and fungal communities, we first evaluated relative abundances at the phylum level. At this level, forest composition appeared not to be a driver of bacterial structure. However, slight differences were observed in Acidobacteriota, Actinobacteriota, Nitrospirota, Planctomycetota, Proteobacteria, RCP2-54, and Verrucomicrobiota. In contrast, soil depth resulted in certain patterns. Proteobacteria showed the greatest relative sequence abundances, which decreased with soil depth (Supplementary Table 3). Similar trends were found for Actinobacteriota and Bacteroidota. Conversely, Chloroflexi, Gemmatimonadota, and Methylomirabilota increased in abundance along with deeper soil layers. The relative abundances of Acidobacteriota, Verrucomicrobiota, Nitrospirota, and Planctomycetota slowly differed across soil depths, and Firmicutes did not show differences across depth (Figure 1A; Supplementary Table 3). For fungi, the greatest relative abundances of reads were affiliated to Ascomycota and Basidiomycota, followed by Mortierellomycota, Mucoromycota, and unclassified fungi. Other fungal phyla were scarcely detected. For purely deciduous forest plots, we detected an increasing relative sequence abundance of Basidiomycota with soil depth and decreasing relative sequence abundances of Ascomycota and Mortierellomycota (Supplementary Table 4). In mixed forests, the Mucoromycota increased in relative sequence abundance with soil depth. Furthermore, Ascomycota and Mortierellomycota showed higher relative sequence abundances in mixed forest plots compared with either deciduous or evergreen ones, particularly at 20–30 cm (Figure 1B; Supplementary Table 4).

**Figure 2:**
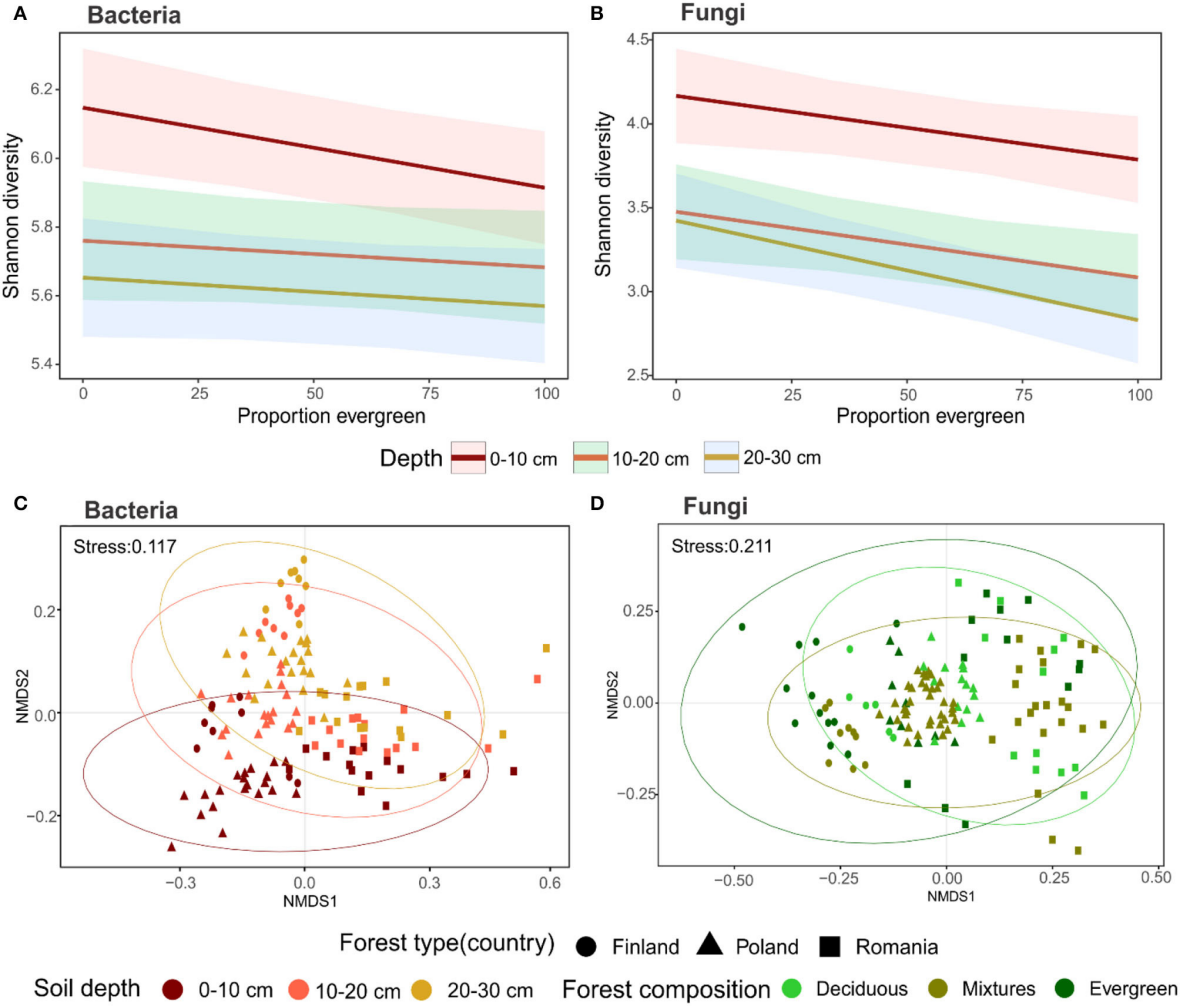
Diversity and community composition associated with forest compositions and soil depth: **(A)** Bacterial and **(B)** Fungal Shannon diversity in relation to evergreen proportions, lines represents linear model estimates and shaded areas represent 95% confidence; **(C)** Bacterial and **(D)** Fungal community composition depicted as NMDS scaling based on Bray-Curtis dissimilarity, and different colors represent the strongest impacting variables according to PERMANOVA (for statistical details see Supplementary Tables 5, 6).

In a second step, we analyzed microbial Shannon diversity and ASV richness in relation to forest composition and sampling depth. Our results revealed general patterns of Shannon diversity in bacteria and fungi in relation to forest composition. With more evergreen trees present, the microbial diversity decreased (bacterial *P* = 0.0041; ANOVA, *P* = 0.005624; PseudoR^2^ = 0.54; fungal *P* = 0.0292; ANOVA, *P* = 7.567e-06; PseudoR^2^ = 0.52; Figures 2A,B). Moreover, bacterial Shannon diversity and ASV richness differed along with soil depth, whereby the topsoil harbored the highest diversities (Figure 2A; Supplementary Table 5). In the upper 20 cm, the bacterial evenness was higher than at lower depths (Supplementary Table 5). Fungi also displayed the highest Shannon diversity, ASV richness, and evenness in the topsoil, but the values were similar in the 20–30 cm soil layer (Figure 2B; Supplementary Table 5).

The forest type was the greatest driver of microbial community composition (Supplementary Table 6). Additionally, soil depth impacted bacterial communities more than forest composition (Figures 2C,D). Conversely, fungal communities were shaped by forest composition, which was verified by PERMANOVA performed individually for each forest type (Figures 2C,D; Supplementary Table 6).

### Bacterial Pathways and Fungal Guilds Highly Associated With Forest Composition and Soil Depth

**Figure 3:**
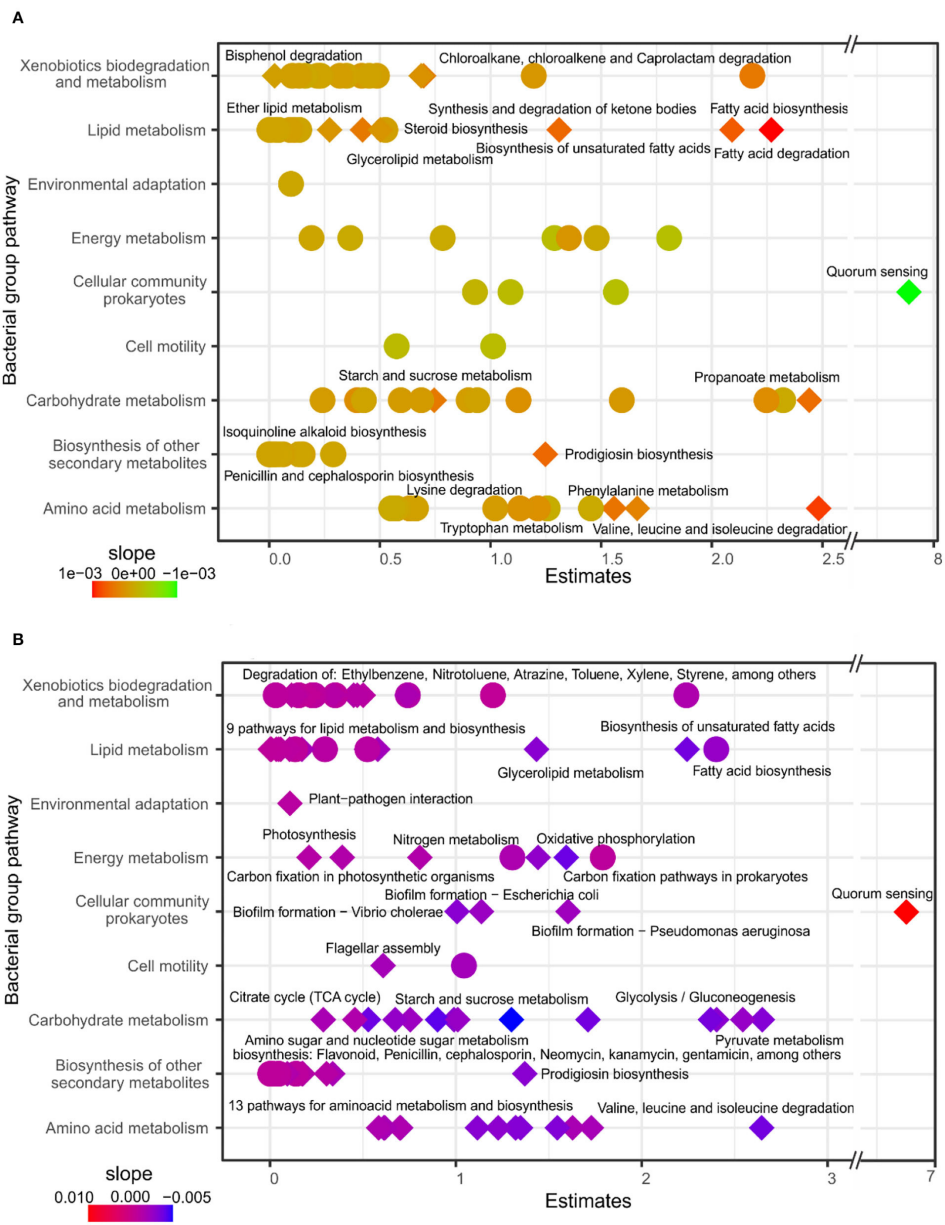
GLM of bacterial pathways related to evergreen tree proportion **(A)** and soil depth **(B)**. Estimates are the intercept of the model and the color represents the slope. Diamonds represent specific pathways that respond with significant differences according to ANOVA (*p* < 0.05) of the GLM. Pathways with significant effects in relation to evergreen tree proportion and soil depth were labeled with specific pathways (Level 2), and model details are provided in Supplementary Tables 7, 8.

We used GLM models to estimate microbial roles at the functional level using bacterial pathways and fungal guilds. In general, of the 96 bacterial pathways, 22 responded to forest composition and 77 to soil depth (Supplementary Tables 7, 8). The models with bacterial pathway abundances using evergreen tree proportion as an explanatory variable did not show a positive association with functional core pathways linked to energy metabolisms, such as carbon fixation, degradation, photosynthesis, or nitrogen. However, significant responses were detected in more specific bacterial pathways related to metabolisms of xenobiotics, lipids, amino acids, biosynthesis of secondary metabolites, and the pathways of carbohydrate degradation like starch, sucrose, and propanoate metabolism. For most of these positive response pathways, with the exception of quorum sensing, the results suggested an increasing bacterial pathway abundance with a higher proportion of evergreen trees (Figure 3A; Supplementary Table 7). Most of the core pathways, such as carbon fixation, nitrogen metabolism, or lipid metabolism, responded to soil depth, and most of the pathways were significantly more represented in the topsoil. In contrast, quorum sensing and atrazine degradation were positively related to deeper soil layers (Figure 3B; Supplementary Table 8).

**Figure 4:**
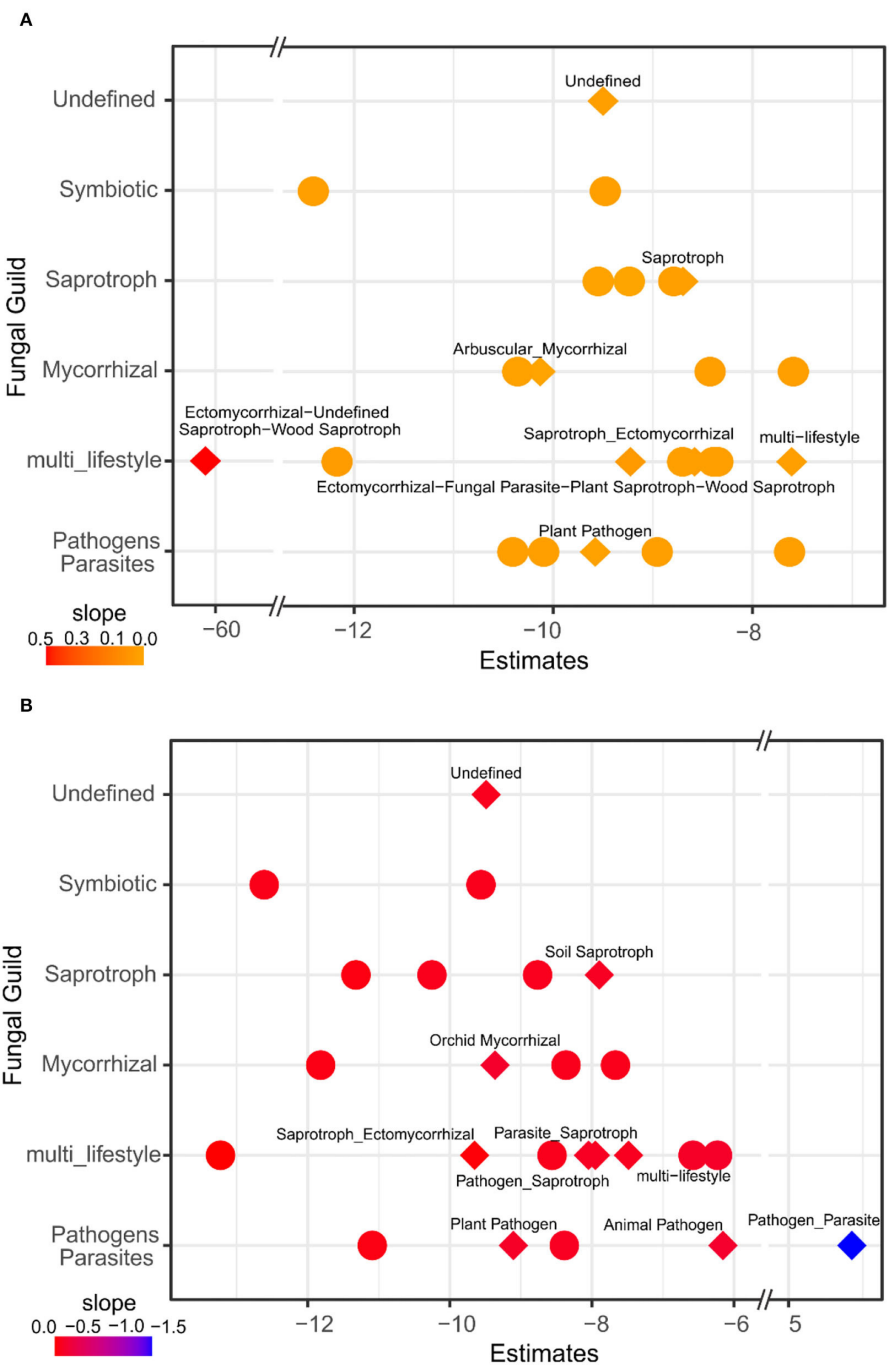
GLM of fungal guilds related to evergreen tree proportion **(A)** and soil depth **(B)**. Estimates are the intercept of the model and the color represents the slope. Diamonds represent specific guilds that respond significantly according to ANOVA (*p* < 0.05) of the GLM. Fungal guilds with significant effects in relation to evergreen tree proportion and soil depth were labeled with specific guilds (Guild 2), and model details are given in Supplementary Tables 9, 10.

Regarding the fungal guilds associated with evergreen tree proportion, the results showed that the saprotrophs, arbuscular mycorrhizal fungi, and plant pathogens had significant responses and showed higher abundances in deciduous forests and mixtures compared to evergreen forests (Figure 4A; Supplementary Table 9). Our results indicated a response of soil saprotrophs, orchid mycorrhizal fungi, and different pathogens/parasites particularly in the topsoil (Figure 4B; Supplementary Table 10).

### Microbial Taxa Closely Associated With Forest Composition and Soil Depth

We used the RF approach to determine microbial taxa indicative of forest composition and soil depth. According to our expectations, the RF model detected microbial indicator features, i.e., microbial taxa with a potential relationship to one or both considered variables. Throughout, the estimated prediction accuracy of our RF models improved from broad to fine taxonomical levels, with ASVs being the best predicting feature for bacteria and fungi. At the bacterial ASV level, the RF validation with the training/test (the mean of the validation performance to classified 29 tested samples) models reached an estimated accuracy of 75% and 84% for forest composition and soil depth, respectively. The fungal ASV-based RF models delivered an estimated accuracy of 81% and 69% for forest composition and soil depth, respectively (Supplementary Figure 2). Moreover, the RF models were used to predict bacterial metabolic pathways and fungal functional guilds. The RF validation estimated accuracy at these functional levels was lower than the ASV taxonomy level. The bacterial pathways displayed an estimated accuracy of 57% and 62% for forest compositions and soil depth, respectively. Concerning fungal guilds, RF reached an estimated accuracy of 58% and 51% for forest composition and soil depth, respectively (Supplementary Figure 2).

We identified the top 10 bacterial and fungal genera associated with forest composition and soil depths by considering the individual mean importance in the best RF model (Tables 1, 2). Most of the microbial genera indicative of soil depth differed from those related to forest composition. For example, the bacterial genus Devosia was highly linked to soil depth, but not to forest composition. Contrastingly, the genus GOUTA6 (Nitrosomonadacea) was highly indicative of forest composition, but less indicative of soil depth. However, the bacterial genera Puia and Caulobacter were comparably associated with both forest composition and soil depth (Tables 1, 2).

**Table 1:** Top 10 bacterial and fungal genera associated with forest composition based on our RF model.

**Deciduous**		**Mixtures**		**Evergreen**	
**Genus**	**Mean decrease**	**Genus**	**Mean decrease**	**Genus**	**Mean decrease**
	**in accuracy**		**in accuracy**		**in accuracy**
**Bacteria**					
GOUTA6	4.796	*Sediminibacterium*	3.969	*Inquilinus*	4.147
*Sediminibacterium*	4.288	*Mycobacterium*	3.707	*Longimycelium*	3.579
*Rhodomicrobium*	3.975	*Stenotrophobacter*	3.544	*Acidocella*	3.252
*Xylophilus*	3.268	*Microlunatus*	3.175	*Actinomadura*	3.043
*Geobacter*	3.209	*Cohnella*	3.135	OLB12	2.926
Unclassified	3.191	mle1-7	3.003	*Micropepsis*	2.668
Candidatus Xiphinematobacter	3.174	*Puia*	2.904	Candidatus Xiphinematobacter	2.592
*Jatrophihabitans*	3.107	*Caulobacter*	2.627	JGI_0001001-H03	2.569
*Pedosphaera*	2.791	*Aquisphaera*	2.614	GOUTA6	2.565
*Pseudolabrys*	2.66	*Anaeromyxobacter*	2.499	*Alicyclobacillus*	2.467
**Fungi**					
*Trichophaea*	7.339	*Trichophaea*	7.41	*Solicoccozyma*	6.998
*Meliniomyces*	6.14	*Sistotrema*	5.236	*Geomyces*	4.999
*Amphinema*	5.52	Unclassified Bionectriaceae	4.778	*Apiotrichum*	4.67
*Hygrophorus*	4.316	*Umbelopsis*	4.43	Unclassified Saccharomycetales	4.126
*Apodus*	4.226	*Trichocladium*	4.199	*Inocybe*	3.682
*Cephalotheca*	3.542	*Solicoccozyma*	3.947	*Mortierella*	3.627
*Tylospora*	3.478	*Inocybe*	3.722	*Oidiodendron*	3.546
*Wilcoxina*	3.428	*Sebacina*	3.716	*Xerocomellus*	3.336
Unclassified	3.409	*Pochonia*	3.022	*Cladophialophora*	3.238
Unclassified bionectriaceae	3.295	Unclassified Saccharomycetales	2.982	*Sistotrema*	3.199

*Genus order is based on the mean decrease in accuracy estimator by each forest composition*.

**Table 2:** Top 10 bacterial and fungal genera associated with soil depth based on our RF model.

**0–10cm**		**10–20cm**		**20–30cm**	
**Genus**	**Mean decrease**	**Genus**	**Mean decrease**	**Genus**	**Mean decrease**
	**in accuracy**		**in accuracy**		**in accuracy**
**Bacteria**					
Devosia	7.661	Devosia	6.831	1921-3	6.796
Ferruginibacter	7.452	Cytophaga	4.969	Rhodoplanes	5.394
Chthoniobacter	7.358	Ferruginibacter	4.841	Caulobacter	5.02
Granulicella	7.342	Pandoraea	4.736	Devosia	4.776
Puia	5.778	Conexibacter	3.789	Mucilaginibacter	4.687
Pandoraea	5.774	Granulicella	3.604	HSB OF53-F07	4.383
Acidipila	5.7	Luteibacter	3.452	Paenibacillus	4.148
Occallatibacter	5.596	Nakamurella	3.338	Occallatibacter	4.004
Phenylobacterium	5.399	Nocardioides	3.276	Edaphobacter	3.92
Pajaroellobacter	5.386	Unclassified	2.964	Granulicella	3.850
**Fungi**					
Tomentella	8.115	Ilyonectria	5.032	Tomentella	8.936
Cortinarius	6.979	Unclassified Agaricomycetes	4.061	Unclassified Hyaloscyphaceae	7.889
Mycena	6.805	Unclassified Mortierellales	3.878	Neobulgaria	6.753
Meliniomyces	6.253	Neobulgaria	3.774	Unclassified Lecanoromycetes	5.867
Unclassified Mortierellales	6.176	Mycena	3.265	Elaphomyces	4.644
Cladophialophora	6.041	Cladosporium	3.107	Exophiala	4.38
Unclassified	5.625	Ascobolus	2.963	Ilyonectria	4.322
Chloridium	5.393	Metarhizium	2.858	Cortinarius	4.103
Unclassified venturiaceae	5.352	Unclassified	2.792	Hygrophorus	4.077
Lactarius	5.245	Cladophialophora	2.738	Unclassified Saccharomycetales	4.053

Genus order is based on the mean decrease accuracy estimator by each soil depth.

In relation to forest composition, deciduous, mixed, and evergreen forests diverged in their associated bacterial communities. For example, the genus GOUTA6 was an important feature in the RF indicating deciduous and evergreen forests of bacterial communities. *Sediminibacterium* was highly associated with deciduous and mixed forests, while *Inquilinus* was linked to evergreen forests (Table 1). Considering the RF importance for the genera associated with soil depth, for some bacteria, importance decreased with depth, e.g., *Devosia* or *Granulicella*, whereas other bacterial genera were more indicative of a particular depth, such as *Chthoniobacter* and *Acidipila* for 0–10 cm, *Cytophaga* and *Conexibacter* for 10–20 cm, and 1921–3 (Ktedonobacteraceae) and *Rhodoplanes* for 20–30 cm (Table 2).

Fungal genera linked to forest composition and soil depths were *Meliniomyces, Cladophialophora*, and *Hygrophorus*, besides unclassified taxa (Tables 1, 2). Fungi associated with forest composition were *Trichophaea* with the highest RF importance in deciduous and mixed forests, and *Sistotrema, Inocybe*, and *Solicoccozyma* with importance in mixed and evergreen forests (Table 2). The fungal genera linked to soil depth appeared to be rather related to the uppermost or lowest depths considered, e.g., *Cortinarius* and, in particular, *Tomentella* showed a high RF importance in 0–10 and 20–30 cm. In contrast, *Mycena* was highly associated with the first 20 cm, while *Ilyonectria* appeared to be more linked to 20–30 cm (Table 2).

## Discussion

### General Bacterial and Fungal Niche Preferences

Our study aimed to investigate bacterial and fungal diversity in relation to forest composition, in terms of the proportion of both evergreen and deciduous trees, and soil depth. In accordance with previous studies, our results showed that an increasing proportion of evergreen trees negatively affected microbial Shannon diversity, which also decreased along with soil depth (Jobbágy and Jackson, 2001; Goebes et al., 2019). In line with Osburn et al. (2019), bacterial communities were shaped by soil depth physicochemical variables, while fungal assemblies were rather shaped by forest composition. The strong link between soil fungi, above- and belowground litter, and also root exudates might be responsible for these patterns (Eisenhauer et al., 2017). The vertical gradient from copiotrophic conditions in the topsoil, i.e., rich in organic compounds, to oligotrophic conditions, i.e., simple minerals, with increasing depth conditions (Jobbágy and Jackson, 2001; Goebes et al., 2019) was apparently a more dominant driver than forest composition for bacterial communities. The bacterial presence in oligotrophic environments is associated with their capability to solubilize key nutrients, such as phosphorus and potassium, from primary minerals (Uroz et al., 2009, 2012; Prada-Salcedo et al., 2014). In contrast, the higher association of fungi with forest composition is perhaps due to their greater prevalence to interact with plants and their wider enzymatic potential to decompose complex organic substrates and recalcitrant compounds from plant and fungal necromass (Fabian et al., 2017; Algora Gallardo et al., 2021). Though we found no interactions between the proportion of evergreen and soil depth affecting microbial diversity, there are reports of such effects. For instance, the different litter types and decay rates between broadleaf and coniferous (needleleaf) trees differentially modify pH and base cation content of soils, and consequently, particular taxa are indirectly selected at surface soil layers (Augusto et al., 2015). Moreover, at deeper soil horizons, the rooting growth system of deep vs. shallow roots, and different rates between the lifespan of short and long roots of broadleaf and coniferous trees affect humus form and soil structure, causing another indirect effect on microbial diversity (Berger and Berger, 2012). These effects can be more pronounced in rhizospheres because the amount and nature of root exudates also change depending on the tree species (Prescott and Grayston, 2013). Therefore, complementary studies on particular microbial groups, e.g., within the rhizosphere or symbionts, could extend the knowledge on how tree–soil interactions affect the microbial communities.

### Potential Bacterial Predicted Pathways and Fungal Guilds

The pathway predictions showed the impact of the proportion of evergreen trees on bacterial pathways, suggesting that the forest composition and mixed forest might affect the microbial processes. By using the same soil samples as this study, Gillespie et al. (2020) experimentally tested microbial responses to drought. Thereby, they found a less decrease in microbial respiration and denitrification in soils obtained from mixed compared to monospecific forests. Likewise, soil microbial stress levels were lower in mixed than in monospecific forests. This enhancement of microbial functional resistance in forest mixtures could be attributed to a higher number of microbial interactions, which primarily carry positive plant–microbe feedbacks (Prada-Salcedo et al., 2021a). Our results revealed that forest composition has a greater relationship with specific pathways, like xenobiotic metabolism, suggesting that more diverse forests could take up xenobiotics at enhanced rates and through different paths (Salem et al., 2017; Desai et al., 2019). Similarly, the higher response of secondary metabolism biosynthesis in top soils with higher evergreen proportions could induce more heterogeneous microenvironments with greater opportunities for interactions and competition that trigger the production of secondary metabolites (Sharrar et al., 2020).

Responses of the fungal guilds, particularly with regard to saprotrophic and root-associated fungi, have been noticed before (Bödeker et al., 2016; Peršoh et al., 2018). Additionally, fungal taxa with multiple lifestyles differ across depths. This group supposedly combines different lifestyles (i.e., biotrophic, necrotrophic, and saprotrophic) and often cope with limited nutritional dependencies, thus several taxa with undefined functions can adapt depending on the niche conditions (Lewis, 1973; Suzuki and Sasaki, 2019). Our results suggest higher responses and abundances (Supplementary Figure 3) of potentially pathogenic fungi in deciduous and mixtures forest, due to the higher number of potential niches and hosts in more diverse forests. Our results are consistent with those of (Nguyen D. et al., 2016), which used the same platform and plots to evaluate the incidence of foliar fungal disease in relation to tree species diversity. The authors found a decrease in the disease incidence with tree species richness in conifers, but not in broadleaved trees for which the incidence tended to increase with tree diversity. These results could suggest that deciduous and mixture forests favor conditions to maintain higher overall pathogen loads. But this does not necessarily mean higher disease levels, as forests dominated by one tree species have shown to be more susceptible to pests and diseases because the huge nutrient source and homogeneous habitat of monocultures normally rise to rapid infection rates (Liu et al., 2018). Fungal saprotrophs were predominantly linked to deciduous forests. In accordance, Chen et al. (2019) evaluated differences between fungi associated with subtropical evergreen and deciduous forests, finding higher abundances of saprophytic fungi in deciduous forests and deducing that these forests mainly harbor fast-growing copiotrophic fungi. Our results also show that within the subsoil, symbiotrophs, mainly mycorrhizal fungi were found (Supplementary Figure 3). Fungi of this guild receive photo-assimilated carbon from plants in exchange for the supply of poorly soluble nutrients that mycorrhizal fungi mobilize from rock weathering. Therefore, it is evident that this guild also responds in the greatest depth (Thorley et al., 2015). The structural and functional diversity of mycorrhizal fungi concerning short- to long-distance extension of their extra-radical mycelium and rhizomorphs enable them an optimal soil exploration (Agerer, 2006) and their dominance in the subsoil.

### Random Forest Modeling Related to Microbial Communities in Forest Ecosystem

The RF approach contributes to identifying indicator species for a particular class of samples or conditions. Thus, we could show the need to study microbial abundances with different methods that may reveal the role of less abundant or unknown microbes, which also participate in the biogeochemical cycles (Jousset et al., 2017). The results of the RF using fine taxonomic ranks, such as genera or ASV, increased classifier accuracy. Functional predictions either from bacterial pathways or fungal guilds did not offer such high levels of accuracy compared to the taxonomical output. The lack of high accuracy values by using functional features, such as RF features, could be explained by the high functional redundancy in soils (Louca et al., 2018). Hence, the model accuracy is probably affected, especially, at broad functional levels like bacterial pathways and fungal guilds because these can be potentially performed by a wide range of extant taxa that cover “core” functions (e.g., respiration, nitrogen, and phosphorus cycling) (Jia and Whalen, 2020).

### Indicator Taxa and Community Structure According to Forest Composition and Soil Depth

Based on the literature, we characterized some of the top indicator taxa from this study to evaluate whether specific conditions of the forest composition or soil depth favor particular microbes and lifestyle strategies. We found bacterial genera, such as *Sediminibacterium, Rhodomicrobium*, and *Jatrophihabitans*, that were indicator taxa within deciduous forests and are reported to display microbial traits common to r-strategists, i.e., higher performances in resource-rich environments (Duchow and Douglas, 1949; Qu and Yuan, 2008; Kim et al., 2015; Papp et al., 2020). These taxa are involved in the fast turnover of litter and consequently fast degradation of cellulose and hemicellulose compounds (Vesterdal et al., 2008; Adams et al., 2019). In contrast, linked to evergreen plots, we found bacterial indicator genera like *Inquilinus, Acidocella*, or *Alicyclobacillus* that could be adapted to typically acidic soils (Jung et al., 2011; Okamoto et al., 2017), and *Longimycelium* and *Actinomadura* (Zakharova et al., 2003; Xia et al., 2013), characterized by slow growth and efficient use of recalcitrant carbon resources and secondary metabolite production, which are traits rather associated with K-strategists and environments where resource supply rates are lower (Ho et al., 2017). Concerning fungi, interestingly, our RF revealed that free-living unicellular fungi were indicators of evergreen forest plots. Yeasts like *Solicoccozyma, Apiotrichum*, and unclassified Saccharomycetales are commonly isolated from forest soils (Liu et al., 2015; Yurkov et al., 2015; James et al., 2016; Mašínová et al., 2016). However, our results showed their clear niche preference for the evergreen forests. According to Birkhofer et al. (2012) and Yarwood et al. (2010), these yeasts can tolerate low pH values, and sandy and drained soils, which are common in evergreen forests (Retallack, 2005; Adams et al., 2019).

The link of the bacterial phyla Bacteroidota and Proteobacteria with the first soil centimeters corresponds to previous studies (Feng et al., 2019). This link is likely due to the copiotrophic properties and associations to aerobic niches in genera like *Devosia, Ferruginibacter*, and *Granulicella* (Nakagawa et al., 1996; Lim et al., 2009; Pankratov and Dedysh, 2010). In general, there were fewer bacterial and fungal taxa of importance for 10–20 cm compared to that in the 0–10 and 20–30 cm, which is probably related to the changing conditions within this intermediate soil compartment, with more microaerophilic and mixing of copiotrophic/oligotrophic conditions (Fierer et al., 2003; Moll et al., 2015; Neira Román et al., 2015) acting as filters for soil microbes, and hence it is complicated to find a clear microbial-niche association or clear indicator taxa. Accordingly, the intermediate depth (10–20 cm) could be seen as a transition zone for soil microbial communities (Eilers et al., 2012; Feng et al., 2019).

## Conclusion

Our analyses provide a comprehensive understanding of bacteria and fungi related to forest composition in terms of evergreen and deciduous trees, and to soil depth. Microbial diversity and functionality of bacterial pathways and fungal guilds were reduced across the vertical gradient. We demonstrate that bacterial community composition varied primarily with soil depth, whereas fungal communities were strongly influenced by tree composition. The microbes within the intermediate soil depth were less predictable compared to topsoil and subsoil. A higher proportion of evergreen trees decrease taxonomic microbial diversity, while increasing the prediction of bacterial pathways abundances. The fungal guilds, especially saprotrophs, arbuscular mycorrhizal fungi, and plant pathogens, were highly represented in deciduous and mixed forests. Likewise, we emphasized the potential impact of forest composition by identifying several microbial functional pathways and guilds, which can be critical in providing higher biosynthetic capacities that support resistance against disturbances, and maintaining forest soil functionality.

Finally, the RF classifier performed with higher accuracy at finer taxonomic resolution compared to predicted bacterial metabolic pathways, and fungal guilds revealed microbial taxa linked to forest composition and soil depth. The identification of microbial indicator taxa by random forest approach can be linked to their functionality, and consequently contributes to explaining plant–microbial interactions under different forest compositions and provides support for forest management decisions.

## Data Availability Statement

The datasets presented in this study can be found in online repositories. The names of the repository/repositories and accession number(s) can be found below: https://www.ebi.ac.uk/ena, PRJEB33611 and experiment accession ERX5307385–ERX5307648.

## Author Contributions

FB conceptualized the study and acquired funding. LP-S together with SoilforEurope members did the sampling work, performed molecular data set analysis, analyzed the data, and wrote the manuscript. JP-S contributed to the machine learning method. KG and AH-B provided substantial contributions to ecological significance. FB, KG, and AH-B provided revisions for the data analyses. All authors contributed to the completion of the manuscript.

## Funding

This research was part of the SoilForEUROPE project funded through the 2015–2016 BiodivERsA COFUND call for research proposals, with the national funders French National Research Agency (ANR, France), Belgian Science Policy Office (Belpo, Belgium), German Research Foundation (DFG, Germany), Research Foundation Flanders (FWO, Belgium), and the Swedish Research Council (Formas, Sweden). LP-S and FB are grateful to the DFG Grant BU 941 28-1 that supported this project.

## Conflict of Interest

The authors declare that the research was conducted in the absence of any commercial or financial relationships that could be construed as a potential conflict of interest.

## Publisher's Note

All claims expressed in this article are solely those of the authors and do not necessarily represent those of their affiliated organizations, or those of the publisher, the editors and the reviewers. Any product that may be evaluated in this article, or claim that may be made by its manufacturer, is not guaranteed or endorsed by the publisher.
