# Supplementary material for: Effects of Tree Composition and Soil Depth on Structure and Functionality of Belowground Microbial Communities in Temperate European Forests
Source: Front Microbiol. 2022 Jul 11;13:920618. doi: 10.3389/fmicb.2022.920618 (PMC9328770; doi:10.3389/fmicb.2022.920618)
Supplement: Supplementary file 1 [file Data_Sheet_1.pdf]

Supplementary material

Effects of tree composition and soil depth on structure and functionality of belowground microbial communities in temperate European forests

Luis Daniel Prada-Salcedo[1,2,5], Juan Pablo Prada-Salcedo[3], Anna Heintz-Buschart[4,5,1], François Buscot[1,5] and Kezia Goldmann[1]

[1] Helmholtz-Centre for Environmental Research - UFZ, Department Soil Ecology, Theodor-Lieser-Straße 4, 06120 Halle (Saale), Germany
[2] University of Leipzig, Department of Biology, Johannisallee 21, 04103 Leipzig, Germany
[3] Department of Bioinformatics, Biocenter, University of Würzburg, Am Hubland, 97074 Würzburg, Germany
[4] Swammerdam Institute for Life Sciences, University of Amsterdam, Science Park 904, 1098 XH, Amsterdam, The Netherlands
[5] German Centre for Integrative Biodiversity Research (iDiv) Halle-Jena-Leipzig, Puschstr. 4, 04103 Leipzig, GermanyLeipzig, Germany

Table S1. Study samples and general location parameters. Each of the plots include the three soil depths 0-10 cm, 10-20 cm and 20-30 cm. More information regarding each plot is storage at https://data.botanik.uni-halle.de/fundiveurope.

Tree type	Country	Forest system	Plot	Evergreen proportion	Latitude	Longitude	Altitude	Annual mean temperature	Annual mean precipitation	Soil type	Composition
Deciduous	Finland	monocultures	FIN07	0	62.67	29.58	120	2.3	625	podzol	*Betula pendula*
			FIN11	0	62.70	29.77	124	2.2	623	podzol	*Betula pendula*
	Poland		POL02	100	52.86	23.88	157	6.9	585	Luvisols	*Carpinus betulus*
			POL12	100	52.87	23.61	160	6.8	583	Luvisols	*Carpinus betulus*
		mixtures	POL13	0	52.90	23.65	160	6.9	578	Luvisols	*Betula pendula, Carpinus betulus, Quercus robur*
		monocultures	POL20	0	52.71	23.62	173	7	576	Luvisols	*Quercus robur*
		mixtures	POL36	0	52.90	23.64	160	6.9	578	Luvisols	*Betula pendula, Carpinus betulus, Quercus robur*
	Romania	monocultures	ROM13	0	47.29	26.04	812	5.7	681	Eutric cambisol	*Fagus sylvatica*
			ROM15	0	47.29	26.04	930	5.2	709	Eutric cambisol	*Acer pseudoplatanus*
			ROM16	0	47.29	26.04	972	5.2	709	Eutric cambisol	*Acer pseudoplatanus*
			ROM26	0	47.29	26.05	782	5.9	671	Eutric cambisol	*Fagus sylvatica*
Evergreen	Finland	monocultures	FIN10	100	62.70	29.74	134	2.1	624	podzol	*Picea abies*
			FIN16	100	62.61	30.34	143	1.8	634	podzol	*Pinus sylvestris*
			FIN21	100	62.41	30.22	135	2	637	podzol	*Pinus sylvestris*
			FIN23	100	62.36	30.21	122	2.1	634	podzol	*Picea abies*
	Poland		POL03	100	52.76	23.69	163	6.9	580	cambisol	*Picea abies*
			POL21	100	52.72	23.58	170	6.9	577	cambisol	*Pinus sylvestris*
			POL40	100	52.78	23.62	175	6.9	580	cambisol	*Pinus sylvestris*
	Romania		ROM01	100	47.30	26.05	838	5.8	675	Eutric cambisol	*Picea abies*
			ROM02	100	47.31	26.05	865	5.8	675	Eutric cambisol	*Picea abies*
			ROM07	100	47.31	26.04	1062	5.2	709	Eutric cambisol	*Abies alba*
			ROM23	100	47.29	26.06	894	5.6	688	Eutric cambisol	*Abies alba*
Mixtures	Finland	mixtures	FIN12	67	63.02	29.79	233	1.4	639	podzol	*Betula pendula, Picea abies, Pinus sylvestris*
			FIN25	67	62.33	30.37	136	2.1	635	podzol	*Betula pendula, Picea abies, Pinus sylvestris*
			FIN26	67	62.33	30.32	108	2.1	636	podzol	*Betula pendula, Picea abies, Pinus sylvestris*
	Poland		POL04	33	52.76	23.66	171	6.8	582	cambisol	*Betula pendula, Picea abies, Quercus robur*
			POL06	33	52.82	23.73	190	6.8	585	Luvisols	*Betula pendula, Carpinus betulus, Picea abies*
			POL10	33	52.89	23.63	145	6.8	582	Luvisols	*Carpinus betulus, Picea abies, Quercus robur*
			POL18	33	52.77	23.75	160	6.9	581	cambisol	*Carpinus betulus, Pinus sylvestris, Quercus robur*

Tree type	Country	Forest system	Plot	Evergreen proportion	Latitude	Longitude	Altitude	Annual mean temperature	Annual mean precipitation	Soil type	Composition
			POL22	67	52.74	23.78	160	6.9	581	Luvisols	*Picea abies, Pinus sylvestris, Quercus robur*
			POL23	67	52.69	23.67	170	6.9	582	cambisol	*Carpinus betulus, Picea abies, Pinus sylvestris*
			POL24	33	52.77	23.75	170	6.9	581	cambisol	*Carpinus betulus, Pinus sylvestris, Quercus robur*
			POL29	33	52.88	23.63	155	6.8	582	Luvisols	*Carpinus betulus, Pinus sylvestris, Quercus robur*
			POL30	67	52.89	23.60	140	6.9	576	cambisol	*Betula pendula, Picea abies, Pinus sylvestris*
			POL33	33	52.68	23.71	184	6.9	582	Luvisols	*Betula pendula, Carpinus betulus, Pinus sylvestris*
			POL38	33	52.65	23.62	165	7	573	cambisol	*Betula pendula, Carpinus betulus, Pinus sylvestris*
			POL43	67	52.71	23.62	186	7	570	Luvisols	*Picea abies, Pinus sylvestris, Quercus robur*
	Romania		ROM03	33	47.31	26.05	869	5.8	675	Eutric cambisol	*Abies alba, Acer pseudoplatanus, Fagus sylvatica*
			ROM10	67	47.30	26.03	968	5.4	701	Eutric cambisol	*Abies alba, Acer pseudoplatanus, Picea abies*
			ROM11	67	47.30	26.04	805	5.8	678	Eutric cambisol	*Abies alba, Fagus sylvatica, Picea abies*
			ROM18	67	47.28	26.05	1012	4.6	742	Eutric cambisol	*Abies alba, Fagus sylvatica, Picea abies*
			ROM20	33	47.29	26.05	869	5.7	681	Eutric cambisol	*Abies alba, Acer pseudoplatanus, Fagus sylvatica*
			ROM22	67	47.29	26.06	843	6.2	655	Eutric cambisol	*Abies alba, Fagus sylvatica, Picea abies*
			ROM25	33	47.28	26.05	1030	4.6	742	Eutric cambisol	*Abies alba, Acer pseudoplatanus, Fagus sylvatica*

Table S2. Specific pathways used as features for the random forest model to estimate functionality from 16S rRNA bacterial communities under forest composition and soil depths. There are 103 Kegg pathways associate to 9 functional groups

Pathway ID – level 1	General Pathway - level 2	Functional Pathway Group level 3
**ko00220**	Arginine biosynthesis	**Amino Acid Metabolism**
**ko00250**	Alanine, aspartate and glutamate metabolism	
**ko00260**	Glycine, serine and threonine metabolism	
**ko00270**	Cysteine and methionine metabolism	
**ko00280**	Valine, leucine and isoleucine degradation	
**ko00290**	Valine, leucine and isoleucine biosynthesis	
**ko00300**	Lysine biosynthesis	
**ko00310**	Lysine degradation	
**ko00330**	Arginine and proline metabolism	
**ko00340**	Histidine metabolism	
**ko00350**	Tyrosine metabolism	
**ko00360**	Phenylalanine metabolism	
**ko00380**	Tryptophan metabolism	
**ko00400**	Phenylalanine, tyrosine and tryptophan biosynthesis	
**ko00232**	Caffeine metabolism	**Biosynthesis Of Other Secondary Metabolites**
**ko00261**	Monobactam biosynthesis	
**ko00311**	Penicillin and cephalosporin biosynthesis	
**ko00331**	Clavulanic acid biosynthesis	
**ko00332**	Carbapenem biosynthesis	
**ko00333**	Prodigiosin biosynthesis	
**ko00401**	Novobiocin biosynthesis	
**ko00404**	Staurosporine biosynthesis	
**ko00405**	Phenazine biosynthesis	
**ko00521**	Streptomycin biosynthesis	
**ko00524**	Neomycin, kanamycin and gentamicin biosynthesis	
**ko00901**	Indole alkaloid biosynthesis	
**ko00940**	Phenylpropanoid biosynthesis	
**ko00941**	Flavonoid biosynthesis	
**ko00943**	Isoflavonoid biosynthesis	
**ko00944**	Flavone and flavonol biosynthesis	
**ko00945**	Stilbenoid, diarylheptanoid and gingerol biosynthesis	
**ko00950**	Isoquinoline alkaloid biosynthesis	
**ko00960**	Tropane, piperidine and pyridine alkaloid biosynthesis	
**ko00965**	Betalain biosynthesis	
**ko00966**	Glucosinolate biosynthesis	

**ko00010**	Glycolysis / Gluconeogenesis	**Carbohydrate Metabolism**
**ko00020**	Citrate cycle (TCA cycle)	
**ko00030**	Pentose phosphate pathway	
**ko00040**	Pentose and glucuronate interconversions	
**ko00051**	Fructose and mannose metabolism	
**ko00052**	Galactose metabolism	
**ko00053**	Ascorbate and aldarate metabolism	
**ko00500**	Starch and sucrose metabolism	
**ko00520**	Amino sugar and nucleotide sugar metabolism	
**ko00562**	Inositol phosphate metabolism	
**ko00620**	Pyruvate metabolism	
**ko00630**	Glyoxylate and dicarboxylate metabolism	
**ko00640**	Propanoate metabolism	
**ko00650**	Butanoate metabolism	
**ko02030**	Bacterial chemotaxis	**Cell Motility**
**ko02040**	Flagellar assembly	
**ko02024**	Quorum sensing	**Cellular Community - Prokaryotes**
**ko02025**	Biofilm formation - Pseudomonas aeruginosa	
**ko02026**	Biofilm formation - Escherichia coli	
**ko05111**	Biofilm formation - Vibrio cholerae	
**ko00190**	Oxidative phosphorylation	**Energy Metabolism**
**ko00195**	Photosynthesis	
**ko00680**	Methane metabolism	
**ko00710**	Carbon fixation in photosynthetic organisms	
**ko00720**	Carbon fixation pathways in prokaryotes	
**ko00910**	Nitrogen metabolism	
**ko00920**	Sulfur metabolism	
**ko04626**	Plant-pathogen interaction	**Environmental Adaptation**
**ko00061**	Fatty acid biosynthesis	**Lipid Metabolism**
**ko00062**	Fatty acid elongation	
**ko00071**	Fatty acid degradation	
**ko00072**	Synthesis and degradation of ketone bodies	
**ko00100**	Steroid biosynthesis	
**ko00120**	Primary bile acid biosynthesis	
**ko00121**	Secondary bile acid biosynthesis	
**ko00140**	Steroid hormone biosynthesis	
**ko00561**	Glycerolipid metabolism	
**ko00564**	Glycerophospholipid metabolism	
**ko00565**	Ether lipid metabolism	
**ko00590**	Arachidonic acid metabolism	
**ko00591**	Linoleic acid metabolism	

**ko00592**	alpha-Linolenic acid metabolism	
**ko00600**	Sphingolipid metabolism	
**ko01040**	Biosynthesis of unsaturated fatty acids	
**ko00361**	Chlorocyclohexane and chlorobenzene degradation	**Xenobiotics Biodegradation And Metabolism**
**ko00362**	Benzoate degradation	
**ko00363**	Bisphenol degradation	
**ko00364**	Fluorobenzoate degradation	
**ko00365**	Furfural degradation	
**ko00621**	Dioxin degradation	
**ko00622**	Xylene degradation	
**ko00623**	Toluene degradation	
**ko00624**	Polycyclic aromatic hydrocarbon degradation	
**ko00625**	Chloroalkane and chloroalkene degradation	
**ko00626**	Naphthalene degradation	
**ko00627**	Aminobenzoate degradation	
**ko00633**	Nitrotoluene degradation	
**ko00642**	Ethylbenzene degradation	
**ko00643**	Styrene degradation	
**ko00791**	Atrazine degradation	
**ko00930**	Caprolactam degradation	

Table S3. Bacterial relative abundances and standard deviation of main phylum across forest compositions (a) and soil depths (b) according to Kruskal-Wallis test ($p > 0.05$) and Dunn post hoc test. Same letter represent no statistical differences, in bold phyla abundances with significant differences.

a

	DECIDUOUS			MIXTURES			EVERGREEN		
**PHYLUM**	Mean Abundances		sd	Mean Abundances		sd	Mean Abundances		sd
**ACIDOBACTERIOTA**	**0.001546313**	**a**	**0.00249**	**0.001603487**	**a**	**0.002572**	**0.001541858**	**b**	**0.003049**
**ACTINOBACTERIOTA**	**0.001370404**	**a**	**0.002355**	**0.001794179**	**b**	**0.002923**	**0.001764115**	**ab**	**0.003054**
BACTEROIDOTA	0.001577576	a	0.002168	0.001379231	a	0.001748	0.001186875	a	0.001986
CHLOROFLEXI	0.002185939	a	0.007426	0.002140455	a	0.005717	0.00220695	a	0.006157
DESULFOBACTEROTA	0.000803636	a	0.000896	0.000716308	a	0.001273	0.00091875	a	0.00209
FIRMICUTES	0.001070303	a	0.001788	0.002042769	a	0.003543	0.001175	a	0.002682
GEMMATIMONADOTA	0.001606303	a	0.002926	0.001354708	a	0.002958	0.00151975	a	0.003142
METHYLOMIRABILOTA	0.003535584	a	0.005674	0.002572835	a	0.005194	0.0038	a	0.007344
MYXOCOCCOTA	0.002959192	a	0.004679	0.002339897	a	0.003972	0.003320417	a	0.005075
**NITROSPIROTA**	**0.003490909**	**a**	**0.002915**	**0.003259692**	**a**	**0.00286**	**0.00168375**	**b**	**0.001575**
**PLANCTOMYCETOTA**	**0.001822424**	**a**	**0.001446**	**0.001658615**	**a**	**0.00154**	**0.00125625**	**b**	**0.001381**
**PROTEOBACTERIA**	**0.00216632**	**a**	**0.004131**	**0.002166259**	**a**	**0.003868**	**0.002178214**	**b**	**0.004549**
**RCP2-54**	**0.001497778**	**a**	**0.002456**	**0.001953026**	**b**	**0.002556**	**0.001827708**	**ab**	**0.003313**
**VERRUCOMICROBIOTA**	**0.002214609**	**ab**	**0.00356**	**0.00253804**	**a**	**0.00404**	**0.002670263**	**b**	**0.006074**
WPS-2	0.001316364	a	0.001306	0.002057846	a	0.002053	0.00137625	a	0.001744

b

	10 CM			20 CM			30 CM		
**PHYLUM**	Mean abundances		sd	Mean abundances		sd	Mean abundances		sd
**ACIDOBACTERIOTA**	**0.001295202**	**a**	**0.0023**	**0.001869044**	**b**	**0.0029**	**0.001563643**	**c**	**0.0027**
**ACTINOBACTERIOTA**	**0.001889886**	**a**	**0.0029**	**0.001642984**	**a**	**0.0028**	**0.001499845**	**b**	**0.00282**
**BACTEROIDOTA**	**0.002271364**	**a**	**0.0026**	**0.001281395**	**b**	**0.0015**	**0.000573256**	**c**	**0.00086**
**CHLOROFLEXI**	**0.000484909**	**a**	**0.0011**	**0.002478363**	**b**	**0.0056**	**0.003580986**	**b**	**0.0091**
**DESULFOBACTEROTA**	**0.001296364**	**a**	**0.0019**	**0.000787907**	**a**	**0.0013**	**0.000268837**	**b**	**0.00059**
FIRMICUTES	0.001545909	a	0.0028	0.001954884	a	0.0038	0.001246977	a	0.0022
**GEMMATIMONADOTA**	**9.52727E-05**	**a**	**0.0003**	**0.00132893**	**b**	**0.0022**	**0.002985116**	**c**	**0.00423**
**METHYLOMIRABILOTA**	**0.001761169**	**a**	**0.005**	**0.003507508**	**b**	**0.0062**	**0.004120797**	**b**	**0.00625**
**MYXOCOCCOTA**	**0.001696364**	**a**	**0.0034**	**0.003026977**	**b**	**0.0048**	**0.003516279**	**b**	**0.00488**
**NITROSPIROTA**	**0.001285455**	**a**	**0.0018**	**0.003783256**	**b**	**0.0029**	**0.00376093**	**b**	**0.00254**
**PLANCTOMYCETOTA**	**0.001607273**	**ab**	**0.0017**	**0.001763256**	**a**	**0.0014**	**0.001432791**	**b**	**0.00142**
**PROTEOBACTERIA**	**0.002349636**	**a**	**0.0043**	**0.002298073**	**a**	**0.0041**	**0.001855748**	**b**	**0.0039**
**RCP2-54**	**0.001813939**	**a**	**0.0034**	**0.001972558**	**b**	**0.0025**	**0.001633178**	**ab**	**0.00207**
**VERRUCOMICROBIOTA**	**0.001692153**	**a**	**0.0037**	**0.002973317**	**b**	**0.0047**	**0.002818507**	**b**	**0.00503**
**WPS-2**	**0.001334545**	**a**	**0.0014**	**0.002356279**	**b**	**0.0022**	**0.001423256**	**b**	**0.00162**

Table S4. Fungi relative abundances and standard deviation of main phylum across forest compositions (a) and soil depths (b) according to Kruskal-Wallis test ($p > 0.05$) and Dunn post hoc test. Same letter represent no statistical differences, in bold phyla abundances with significant differences.

a

	DECIDUOUS			MIXTURES			EVERGREEN		
PHYLUM	Mean Abundances		sd	Mean Abundances		sd	Mean Abundances		sd
**ASCOMYCOTA**	**0.001327348**	**a**	**0.004387**	**0.0025715**	**b**	**0.009106**	**0.001883864**	**a**	**0.009441**
**BASIDIOMYCOTA**	**0.003373866**	**a**	**0.020659**	**0.002955573**	**a**	**0.017799**	**0.003421492**	**b**	**0.024605**
**MORTIERELLOMYCOTA**	**0.002412458**	**a**	**0.006195**	**0.003441346**	**b**	**0.00743**	**0.001910354**	**c**	**0.006218**
**MUCOROMYCOTA**	**0.003508207**	**ab**	**0.011379**	**0.004199038**	**a**	**0.010698**	**0.00242803**	**b**	**0.008157**
**UNCLASSIFIED**	**0.000767045**	**ab**	**0.002122**	**0.001650962**	**a**	**0.005167**	**0.000791667**	**b**	**0.004492**

b

	10 CM			20 CM			30 CM		
	Mean		sd	Mean		sd	Mean		sd
**ASCOMYCOTA**	**0.002132**	**a**	**0.006698**	**0.001866**	**b**	**0.007339**	**0.002252**	**b**	**0.010345**
**BASIDIOMYCOTA**	**0.002493**	**a**	**0.013365**	**0.003534**	**b**	**0.02187**	**0.003515**	**b**	**0.024404**
**MORTIERELLOMYCOTA**	**0.003238**	**a**	**0.007267**	**0.002828**	**ab**	**0.006833**	**0.002324**	**b**	**0.006466**
MUCOROMYCOTA	0.002125	a	0.004557	0.00449	a	0.012026	0.004143	a	0.012343
UNCLASSIFIED	0.000953	a	0.003227	0.001371	a	0.004659	0.001315	a	0.005184

Table S5. Means of Shannon diversity, observed richness and evenness across forest compositions and soil depths, and according to ANOVA (*p* > 0.05) and Tukey HSD post hoc test. Analysis were made by overall (a & d) and between forest composition (b & e) and soil depths (c & f) for bacteria and fungi, respectively. Same letter represent no statistical differences

Bacteria:

a

OVERALL

	Shannon	Observed	Evenness
**DECIDUOUS**	5.86a	686.03a	0.90a
**MIXTURES**	5.85a	661.95a	0.90a
**EVERGREEN**	5.68b	623.59a	0.88b
**10 CM**	6.05a	787.18a	0.90a
**20 CM**	5.74b	603.62b	0.89a
**30 CM**	5.64b	582.06b	0.88b

b

	**DECIDUOUS**			**MIXTURES**			**EVERGREEN**		
	Shannon	Observed	Evenness	Shannon	Observed	Evenness	Shannon	Observed	Evenness
**10 CM**	6.15a	842.27a	0.91a	6.08a	788.90a	0.91a	5.87a	728.63a	0.89a
**20 CM**	5.82b	628.36b	0.90ab	5.75b	600.28b	0.90b	5.65ab	585.27b	0.88a
**30 CM**	5.62b	587.45b	0.88b	5.71b	593.86b	0.89b	5.11b	550.20b	0.87a

c

	**10 CM**			**20 CM**			**30 CM**		
	Shannon	Observed	Evenness	Shannon	Observed	Evenness	Shannon	Observed	Evenness
**DECIDUOUS**	6.15a	842.27a	0.91a	5.82a	628.36a	0.90a	5.62a	587.45a	0.88a
**MIXTURES**	6.08a	788.90a	0.91a	5.75a	600.28a	0.90a	5.71a	593.86a	0.89a
**EVERGREEN**	5.87b	728.63a	0.89b	5.65a	585.27a	0.88a	5.51a	550.20a	0.87a

Fungi:

d

OVERALL			
	Shannon	Observed	Evenness
**DECIDUOUS**	3.51a	115.51ab	0.74ab
**MIXTURES**	3.62a	116.50a	0.74a
**EVERGREEN**	3.15b	92.69b	0.70b
**10 CM**	3.99a	146.94a	0.80a
**20 CM**	3.29b	94.20b	0.72b
**30 CM**	3.13b	89.27b	0.70b

e

	DECIDUOUS			MIXTURES			EVERGREEN		
	Shannon	Observed	Evenness	Shannon	Observed	Evenness	Shannon	Observed	Evenness
**10 CM**	4.15a	160.63a	0.82a	4.05a	152.86a	0.80a	3.73a	121.36a	0.78a
**20 CM**	3.28b	94.54b	0.73b	3.43b	100.57b	0.74b	3.04b	81.72b	0.69b
**30 CM**	3.10b	91.36b	0.69b	3.38b	95.36b	0.74b	2.69b	75.00b	0.63b

f

	10 CM			20 CM			30 CM		
	Shannon	Observed	Evenness	Shannon	Observed	Evenness	Shannon	Observed	Evenness
**DECIDUOUS**	4.15a	160.63a	0.82a	3.28a	94.54a	0.69ab	3.10ab	91.36a	0.69ab
**MIXTURES**	4.05a	152.86a	0.80a	3.43a	100.57a	0.74a	3.38a	95.36a	0.74a
**EVERGREEN**	3.73b	121.36b	0.78a	3.04a	81.72a	0.63b	2.69b	75.00a	0.63b

Table S6. Results of PERMANOVA between forest composition and soil depths for bacteria and fungi.

a Bacteria:

VARIABLE	OVERALL			FINLAND			POLAND			ROMANIA		
	F.Model	R2	Pr(>F)	F.Model	R2	Pr(>F)	F.Model	R2	Pr(>F)	F.Model	R2	Pr(>F)
**DEPTH**	9.94	0.10	0.001 ***	7.29	0.35	0.001 ***	11.89	0.28	0.001 ***	2.56	0.10	0.001 ***
**FOREST COMPOSITION**	2.67	0.02	0.001 ***	2.93	0.14	0.005 **	3.13	0.07	0.002 **	2.03	0.08	0.015 **
**FOREST TYPE**	20.63	0.21	0.001 ***									
**RESIDUALS**		0.64			0.50			0.64			0.81	

b Fungi:

VARIABLE	OVERALL			FINLAND			POLAND			ROMANIA		
	F.Model	R2	Pr(>F)	F.Model	R2	Pr(>F)	F.Model	R2	Pr(>F)	F.Model	R2	Pr(>F)
**DEPTH**	2.61	0.03	0.001 ***	1.37	0.09	0.055.	4.27	0.12	0.001***	0.98	0.04	0.511
**FOREST COMPOSITION**	3.77	0.04	0.001 ***	2.51	0.16	0.001 ***	2.06	0.10	0.001 ***	2.88	0.12	0.001 ***
**FOREST TYPE**	9.91	0.12	0.001 ***									
**RESIDUALS**		0.79			0.73			0.77			0.83	

Table S7. GLM model coefficients for bacterial pathways in relation to evergreen tree proportion. For level 2, in bold bacterial pathways responding to evergreen tree proportion according to according to ANOVA ($p < 0.05$) of the GLM.

LEVEL 3	LEVEL 2	INTERCEPT	SLOPE	PR(>CHISQ)	R2
**AMINO ACID METABOLISM**	**Tryptophan metabolism**	**1.558271**	**0.000532595**	**0.011347378**	**0.04733833**
	**Valine, leucine and isoleucine degradation**	**2.482343**	**0.000968978**	**0.011818091**	**0.0468306**
	**Lysine degradation**	**1.241821**	**0.000587995**	**0.012426808**	**0.0462032**
	**Phenylalanine metabolism**	**1.662546**	**0.000391333**	**0.028563225**	**0.03582938**
	Arginine and proline metabolism	1.214049	0.000232714	0.230128917	0.01104005
	Tyrosine metabolism	1.13345	0.000220954	0.299656754	0.008269961
	Valine, leucine and isoleucine biosynthesis	0.5570447	-8.95013E-05	0.343118188	0.006918819
	Histidine metabolism	0.6336566	0.000103982	0.476099409	0.003920882
	Cysteine and methionine metabolism	1.258511	-0.000123294	0.664140008	0.001459367
	Alanine, aspartate and glutamate metabolism	1.021383	9.20549E-05	0.678393693	0.001331091
	Arginine biosynthesis	0.6632356	3.47306E-05	0.7257232	0.000953159
	Glycine, serine and threonine metabolism	1.453716	-7.41824E-05	0.726931429	0.000944439
	Lysine biosynthesis	0.5772202	-3.08407E-05	0.75161949	0.000775949
	Phenylalanine, tyrosine and tryptophan biosynthesis	0.5513551	-4.95835E-05	0.755871458	0.000748765
**BIOSYNTHESIS OF OTHER SECONDARY METABOLITES**	**Prodigiosin biosynthesis**	**1.247899**	**0.000654967**	**0.002488819**	**0.06622421**
	**Isoquinoline alkaloid biosynthesis**	**0.1169977**	**7.95571E-05**	**0.021298756**	**0.03947948**
	**Penicillin and cephalosporin biosynthesis**	**0.02665266**	**3.43879E-05**	**0.040236111**	**0.03158897**
	Flavone and flavonol biosynthesis	0.001152357	4.87033E-06	0.056425629	0.02743866
	Caffeine metabolism	0.01053142	6.90866E-06	0.140664677	0.01654846
	Indole alkaloid biosynthesis	0.01013079	3.54759E-06	0.232289417	0.01093938
	Streptomycin biosynthesis	0.2901537	9.52586E-05	0.269962213	0.009345474
	Phenylpropanoid biosynthesis	0.06287514	4.74216E-05	0.295102094	0.008426054
	Carbapenem biosynthesis	0.04436472	-1.55391E-05	0.349757061	0.006732187
	Neomycin, kanamycin and gentamicin biosynthesis	0.0285812	2.6039E-05	0.356280409	0.006553373
	Betalain biosynthesis	0.02304346	6.4515E-06	0.358799313	0.006485504
	Glucosinolate biosynthesis	0.03233739	-5.95249E-06	0.457518276	0.004260524
	Tropane, piperidine and pyridine alkaloid biosynthesis	0.151414	2.57672E-05	0.540273947	0.002898799
	Clavulanic acid biosynthesis	2.86828E-05	-7.89854E-08	0.597626414	0.002154964

	Novobiocin biosynthesis	0.1490791	2.44457E-05	0.610141777	0.002011207
	Monobactam biosynthesis	0.288352	-1.64229E-05	0.729663833	0.000924884
	Phenazine biosynthesis	0.1352795	-8.25815E-06	0.798008828	0.000507475
	Staurosporine biosynthesis	0.01933689	9.09174E-07	0.880346718	0.000175622
	Flavonoid biosynthesis	0.002407438	-2.15564E-07	0.947106941	3.41153E-05
	Stilbenoid, diarylheptanoid and gingerol biosynthesis	0.002407438	-2.15564E-07	0.947106941	3.41153E-05
	Isoflavonoid biosynthesis	0.000812674	6.19382E-08	0.983637234	3.26064E-06
**CARBOHYDRATE METABOLISM**	**Butanoate metabolism**	**2.518721**	**0.000650066**	**0.02364794**	**0.0381767**
	**Starch and sucrose metabolism**	**0.7455036**	**0.000479055**	**0.035111636**	**0.03327148**
	**Propanoate metabolism**	**2.440196**	**0.000611841**	**0.03662943**	**0.03274829**
	Galactose metabolism	0.3972946	0.000265753	0.158462701	0.01518324
	Inositol phosphate metabolism	0.241014	9.54347E-05	0.226687306	0.01120274
	Pyruvate metabolism	2.246732	0.000291238	0.22865065	0.01110958
	Fructose and mannose metabolism	0.6872433	0.000102949	0.416916187	0.005082498
	Pentose and glucuronate interconversions	0.5946489	9.97883E-05	0.423863145	0.00493352
	Amino sugar and nucleotide sugar metabolism	1.126504	0.000240099	0.456680558	0.004276348
	Glycolysis / Gluconeogenesis	1.593026	0.000143581	0.577028512	0.002405542
	Citrate cycle (TCA cycle)	0.9011844	7.33607E-05	0.731295647	0.000913315
	Glyoxylate and dicarboxylate metabolism	2.322261	-6.90478E-05	0.76303128	0.000704185
	Ascorbate and aldarate metabolism	0.4278822	9.60299E-06	0.857524417	0.00024978
	Pentose phosphate pathway	0.9404451	1.50286E-05	0.895701928	0.000133202
**CELL MOTILITY**	Flagellar assembly	0.5760657	-0.00030191	0.133283491	0.0171716
	Bacterial chemotaxis	1.012016	-0.000336704	0.275439153	0.009136353
**CELLULAR COMMUNITY - PROKARYOTES**	**Quorum sensing**	**7.668476**	**-0.001339952**	**0.04469827**	**0.03029383**
	Biofilm formation - Escherichia coli	1.090106	-0.000317615	0.098323404	0.02074302
	Biofilm formation - Pseudomonas aeruginosa	1.566599	-0.000378516	0.122360931	0.01816649
	Biofilm formation - Vibrio cholerae	0.9297244	-0.00021213	0.46859952	0.004055398
**ENERGY METABOLISM**	Methane metabolism	1.288905	-0.000332653	0.093501276	0.02134111
	Sulfur metabolism	1.807056	-0.000378183	0.104231943	0.02005147
	Carbon fixation in photosynthetic organisms	0.3665104	-8.71037E-05	0.320027624	0.007607066
	Carbon fixation pathways in prokaryotes	1.353927	0.000176296	0.374421179	0.00607863
	Nitrogen metabolism	0.7841501	-8.3054E-05	0.526573154	0.003098896
	Photosynthesis	0.1914373	-1.86473E-05	0.759336565	0.000727003

	Oxidative phosphorylation	1.479343	-5.92929E-05	0.846995383	0.00028853
**ENVIRONMENTAL ADAPTATION**	Plant-pathogen interaction	0.09874993	-3.45101E-05	0.238983789	0.01063436
**LIPID METABOLISM**	**Steroid biosynthesis**	**0.004432168**	**2.39668E-05**	**0.00072702**	**0.08132003**
	**Biosynthesis of unsaturated fatty acids**	**1.310149**	**0.000662853**	**0.00263077**	**0.06553797**
	**Fatty acid biosynthesis**	**2.091436**	**0.000768115**	**0.004421478**	**0.05909516**
	**Fatty acid degradation**	**2.26881**	**0.001139802**	**0.005263126**	**0.05692609**
	**Synthesis and degradation of ketone bodies**	**0.5027069**	**0.000183715**	**0.006289128**	**0.05470639**
	**Glycerolipid metabolism**	**0.4216904**	**0.000479592**	**0.009997048**	**0.04892102**
	**Steroid hormone biosynthesis**	**0.2729795**	**0.000179107**	**0.01914697**	**0.04080692**
	**Ether lipid metabolism**	**0.04583137**	**4.76311E-05**	**0.036480479**	**0.03279864**
	alpha-Linolenic acid metabolism	0.09391342	5.23538E-05	0.059508793	0.02678985
	Sphingolipid metabolism	0.1133681	0.000148982	0.06104753	0.02647898
	Secondary bile acid biosynthesis	0.04318835	1.81478E-05	0.0949447	0.0211587
	Arachidonic acid metabolism	0.03236493	-1.58981E-05	0.141424316	0.01648638
	Linoleic acid metabolism	0.005113772	6.22156E-06	0.225488703	0.01126009
	Fatty acid elongation	0.00098563	1.30207E-06	0.526993475	0.003092619
	Primary bile acid biosynthesis	0.13581	-1.00718E-05	0.661179484	0.001486887
	Glycerophospholipid metabolism	0.5251883	3.69008E-05	0.772817293	0.000645652
**XENOBIOTICS BIODEGRADATION AND METABOLISM**	**Bisphenol degradation**	**0.02463081**	**5.67687E-05**	**0.004254246**	**0.0595747**
	**Caprolactam degradation**	**0.6976234**	**0.000433727**	**0.013982872**	**0.04472964**
	**Chloroalkane and chloroalkene degradation**	**0.6867476**	**0.000211045**	**0.021704436**	**0.03924444**
	Ethylbenzene degradation	0.1050553	4.99173E-05	0.064703218	0.02577195
	Benzoate degradation	2.183098	0.000492665	0.115184275	0.0188742
	Nitrotoluene degradation	0.1348723	4.09577E-05	0.144223126	0.01626076
	Naphthalene degradation	0.4180057	0.00013162	0.187575313	0.01328289
	Styrene degradation	0.4869893	5.79082E-05	0.248527577	0.01021672
	Xylene degradation	0.3195153	7.35271E-05	0.290046282	0.008602918
	Furfural degradation	0.1627589	-5.1248E-05	0.377731796	0.005995395
	Aminobenzoate degradation	1.195361	0.000161012	0.39911149	0.005481429
	Dioxin degradation	0.2275134	3.30685E-05	0.603250625	0.002089586
	Chlorocyclohexane and chlorobenzene degradation	0.4515895	2.34309E-05	0.696633498	0.00117689
	Toluene degradation	0.2322582	-1.20558E-05	0.727525858	0.000940166
	Atrazine degradation	0.2184949	3.41318E-06	0.833945035	0.000340588

Fluorobenzoate degradation	0.2252089	-5.72945E-06	0.894295782	0.000136838
Polycyclic aromatic hydrocarbon degradation	0.349305	7.20585E-06	0.963921916	1.58601E-05

Table S8. GLM model coefficients for bacterial pathways in relation to depth. For level 2, in bold bacterial pathways responding to soil depth according to according to ANOVA ($p < 0.05$) of the GLM.

LEVEL 3	LEVEL 2	INTERCEPT	SLOPE	PR(>CHISQ)	R2
**AMINO ACID METABOLISM**	**Arginine and proline metabolism**	**1.320657565**	**-0.004716059**	**1.32444E-09**	**0.2218462**
	**Glycine, serine and threonine metabolism**	**1.545693724**	**-0.004819055**	**2.27324E-08**	**0.1949874**
	**Lysine biosynthesis**	**0.617893636**	**-0.00212567**	**9.97963E-08**	**0.1803171**
	**Histidine metabolism**	**0.702727642**	**-0.003183176**	**1.05713E-07**	**0.179736**
	**Alanine, aspartate and glutamate metabolism**	**1.116014602**	**-0.004498687**	**1.09777E-06**	**0.1554749**
	**Phenylalanine, tyrosine and tryptophan biosynthesis**	**0.612057092**	**-0.003182102**	**1.69641E-06**	**0.1508209**
	**Tyrosine metabolism**	**1.227471967**	**-0.004116385**	**4.44628E-06**	**0.1403561**
	**Cysteine and methionine metabolism**	**1.348067317**	**-0.004831492**	**6.77527E-05**	**0.1095624**
	**Valine, leucine and isoleucine biosynthesis**	**0.584263508**	**-0.001610394**	**6.80829E-05**	**0.1095059**
	**Arginine biosynthesis**	**0.698088488**	**-0.00165461**	**9.38139E-05**	**0.1057644**
	**Valine, leucine and isoleucine degradation**	**2.645001105**	**-0.00552048**	**0.001295334**	**0.07426786**
	**Phenylalanine metabolism**	**1.728650838**	**-0.002250249**	**0.004874683**	**0.05788083**
	**Lysine degradation**	**1.321849828**	**-0.002412591**	**0.02399732**	**0.0379942**
	**Tryptophan metabolism**	**1.627689833**	**-0.002031183**	**0.0341095**	**0.03362974**
**BIOSYNTHESIS OF OTHER SECONDARY METABOLITES**	**Phenylpropanoid biosynthesis**	**0.093046016**	**-0.001384981**	**5.68829E-17**	**0.3520266**
	**Flavone and flavonol biosynthesis**	**0.002962407**	**-7.75635E-05**	**3.18369E-16**	**0.340781**
	**Isoquinoline alkaloid biosynthesis**	**0.14209395**	**-0.001042592**	**1.18123E-15**	**0.3319603**
	**Tropane, piperidine and pyridine alkaloid biosynthesis**	**0.176454278**	**-0.001186545**	**9.15691E-14**	**0.3009652**
	**Penicillin and cephalosporin biosynthesis**	**0.037876967**	**-0.000469557**	**4.8804E-13**	**0.2882856**
	**Streptomycin biosynthesis**	**0.337025027**	**-0.002092706**	**1.50291E-09**	**0.2206878**
	**Neomycin, kanamycin and gentamicin biosynthesis**	**0.042916929**	**-0.000648508**	**1.52513E-08**	**0.1988624**
	**Novobiocin biosynthesis**	**0.171530743**	**-0.001060219**	**6.21863E-08**	**0.185061**

	**Glucosinolate biosynthesis**	**0.035435186**	**-0.000171729**	**1.96142E-07**	**0.1734519**
	**Betalain biosynthesis**	**0.026399457**	**-0.000150845**	**1.96727E-07**	**0.1734214**
	**Flavonoid biosynthesis**	**0.003729931**	**-6.69681E-05**	**6.48799E-07**	**0.1610385**
	**Stilbenoid, diarylheptanoid and gingerol biosynthesis**	**0.003729931**	**-6.69681E-05**	**6.48799E-07**	**0.1610385**
	**Caffeine metabolism**	**0.012769355**	**-9.34791E-05**	**2.17393E-06**	**0.1481485**
	**Prodigiosin biosynthesis**	**1.370794049**	**-0.004381414**	**2.94639E-06**	**0.1448524**
	**Monobactam biosynthesis**	**0.304037258**	**-0.000832098**	**3.85453E-05**	**0.1160854**
	**Staurosporine biosynthesis**	**0.020794271**	**-7.06696E-05**	**0.007904873**	**0.05185333**
	**Clavulanic acid biosynthesis**	**5.86013E-05**	**-1.71721E-06**	**0.009336788**	**0.04977446**
	Carbapenem biosynthesis	0.046414896	-0.000145302	0.05065026	0.02875927
	Phenazine biosynthesis	0.139950244	-0.00025697	0.07485825	0.02400747
	Indole alkaloid biosynthesis	0.010490861	-8.39372E-06	0.5338612	0.002991309
	Isoflavonoid biosynthesis	0.000859447	-2.17868E-06	0.873307	0.000197072
**CARBOHYDRATE METABOLISM**	**Galactose metabolism**	**0.52878552**	**-0.005874841**	**9.31798E-18**	**0.3634406**
	**Pentose phosphate pathway**	**1.010050194**	**-0.003452689**	**1.85322E-16**	**0.3443553**
	**Pentose and glucuronate interconversions**	**0.673415333**	**-0.00368126**	**1.87227E-15**	**0.3288065**
	**Starch and sucrose metabolism**	**0.901434201**	**-0.006519556**	**8.35511E-14**	**0.3016469**
	**Ascorbate and aldarate metabolism**	**0.456851536**	**-0.001427858**	**4.74251E-12**	**0.2703234**
	**Inositol phosphate metabolism**	**0.28654944**	**-0.002025177**	**7.87782E-11**	**0.246882**
	**Amino sugar and nucleotide sugar metabolism**	**1.29889032**	**-0.00799749**	**4.22002E-10**	**0.2321809**
	**Fructose and mannose metabolism**	**0.754360095**	**-0.003087907**	**1.07537E-09**	**0.2237482**
	**Pyruvate metabolism**	**2.370026493**	**-0.00539391**	**5.43415E-08**	**0.1864042**
	**Glycolysis / Gluconeogenesis**	**1.710695066**	**-0.005514399**	**1.94107E-07**	**0.1735586**
	**Citrate cycle (TCA cycle)**	**0.989600964**	**-0.004237736**	**2.00074E-06**	**0.1490447**
	**Glyoxylate and dicarboxylate metabolism**	**2.402983246**	**-0.00424007**	**1.1471E-05**	**0.1298444**
	**Butanoate metabolism**	**2.64847687**	**-0.004739182**	**0.000164359**	**0.09916283**
	**Propanoate metabolism**	**2.543598347**	**-0.003520706**	**0.007224031**	**0.05297749**
**CELL MOTILITY**	**Flagellar assembly**	**0.608527078**	**-0.002453077**	**0.0059518**	**0.05539375**
	Bacterial chemotaxis	1.043328466	-0.002490341	0.07235967	0.02441728
**CELLULAR COMMUNITY - PROKARYOTES**	**Biofilm formation - Escherichia coli**	**1.137276522**	**-0.003234225**	**9.89875E-05**	**0.1051356**
	**Quorum sensing**	**7.378555806**	**0.010896**	**0.000182814**	**0.09790191**
	**Biofilm formation - Vibrio cholerae**	**1.006076595**	**-0.004411134**	**0.000505845**	**0.08571586**
	**Biofilm formation - Pseudomonas aeruginosa**	**1.603491733**	**-0.002884514**	**0.008100393**	**0.0515483**

**ENERGY METABOLISM**	**Carbon fixation pathways in prokaryotes**	**1.44207939**	**-0.003943639**	**2.05665E-06**	**0.1487474**
	**Oxidative phosphorylation**	**1.59364654**	**-0.00589903**	**4.73724E-06**	**0.1396598**
	**Photosynthesis**	**0.209755183**	**-0.000970311**	**0.000210475**	**0.09622841**
	**Carbon fixation in photosynthetic organisms**	**0.388379383**	**-0.001335327**	**0.000440464**	**0.08738607**
	**Nitrogen metabolism**	**0.806089567**	**-0.001327815**	**0.02267059**	**0.03870208**
	Methane metabolism	1.304038034	-0.001667183	0.06253569	0.02618598
	Sulfur metabolism	1.790050606	-0.000178322	0.866887	0.000217739
**ENVIRONMENTAL ADAPTATION**	**Plant-pathogen interaction**	**0.106949238**	**-0.000505707**	**5.66606E-05**	**0.1116383**
**LIPID METABOLISM**	**Sphingolipid metabolism**	**0.171354899**	**-0.002504043**	**5.79629E-18**	**0.3663707**
	**Glycerophospholipid metabolism**	**0.579654826**	**-0.00263316**	**6.63861E-07**	**0.160797**
	**Fatty acid biosynthesis**	**2.243872601**	**-0.005555517**	**1.65031E-06**	**0.1511169**
	**Ether lipid metabolism**	**0.057478227**	**-0.000454633**	**2.62816E-06**	**0.1460941**
	**Biosynthesis of unsaturated fatty acids**	**1.433958026**	**-0.004405808**	**3.9983E-06**	**0.1415204**
	**Glycerolipid metabolism**	**0.519619411**	**-0.003606817**	**7.06152E-06**	**0.1352525**
	**alpha-Linolenic acid metabolism**	**0.104842997**	**-0.000405745**	**0.000906497**	**0.07863346**
	**Fatty acid elongation**	**0.001589127**	**-2.67388E-05**	**0.003042192**	**0.0637383**
	**Linoleic acid metabolism**	**0.006685242**	**-6.19017E-05**	**0.006410041**	**0.0544689**
	**Arachidonic acid metabolism**	**0.033811408**	**-0.00011598**	**0.01622961**	**0.04286926**
	**Steroid biosynthesis**	**0.007126595**	**-6.98496E-05**	**0.03383837**	**0.03372851**
	**Secondary bile acid biosynthesis**	**0.046183554**	**-0.000100824**	**0.03921898**	**0.03190479**
	Fatty acid degradation	2.400677603	-0.003508955	0.06170524	0.02634858
	Steroid hormone biosynthesis	0.295659265	-0.000649684	0.06233534	0.026225
	Synthesis and degradation of ketone bodies	0.523582637	-0.00054656	0.0771315	0.02364691
	Primary bile acid biosynthesis	0.135585982	-1.6237E-05	0.8759841	0.000188766
**XENOBIOTICS BIODEGRADATION AND METABOLISM**	**Chloroalkane and chloroalkene degradation**	**0.737392325**	**-0.001966187**	**3.82729E-07**	**0.1665564**
	**Toluene degradation**	**0.245900162**	**-0.000717625**	**5.41569E-07**	**0.1629345**
	**Atrazine degradation**	**0.212311134**	**0.000319697**	**2.61979E-06**	**0.1461287**
	**Nitrotoluene degradation**	**0.145621095**	**-0.000427762**	**0.000466661**	**0.08668934**
	**Ethylbenzene degradation**	**0.115701016**	**-0.000398145**	**0.000802119**	**0.0801242**
	**Chlorocyclohexane and chlorobenzene degradation**	**0.469010276**	**-0.000810471**	**0.002017143**	**0.06881978**
	**Xylene degradation**	**0.341250163**	**-0.000890322**	**0.003602176**	**0.06164215**
	**Naphthalene degradation**	**0.450095781**	**-0.00125158**	**0.004567385**	**0.05869124**
	**Styrene degradation**	**0.500922143**	**-0.000541332**	**0.01527744**	**0.04362401**

Dioxin degradation	0.237379228	-0.000404972	0.1567071	0.01531015
Bisphenol degradation	0.0301068	-0.000119966	0.1925556	0.01299144
Caprolactam degradation	0.741437253	-0.00101575	0.2110467	0.01198055
Benzoate degradation	2.240003187	-0.001512038	0.2877708	0.008683785
Furfural degradation	0.154768311	0.000261247	0.3199115	0.00761069
Fluorobenzoate degradation	0.227320761	-0.000121631	0.532408	0.003012551
Aminobenzoate degradation	1.199143369	0.000249441	0.773339	0.000642609
Polycyclic aromatic hydrocarbon degradation	0.350653137	-4.80042E-05	0.9469	3.43832E-05

Table S9. GLM model coefficients for fungal guilds in relation to evergreen tree proportion. For Guild 2, in bold fungal guilds responding to evergreen tree proportion according to according to ANOVA ($p < 0.05$) of the GLM.

GUILD 1	GUILD 2	INTERCEPT	SLOPE	PR(>CHISQ)	R_VALUE
**PATHOGENS/PARASITES**	**Plant Pathogen**	**-9.578711485**	**-0.013953071**	**2.13647E-05**	**0.001753864**
	Animal Pathogen	-7.627991038	-0.002874909	0.400526621	0.000355019
	Pathogen	-10.09642002	-0.003912754	0.589343538	0.000334268
	Pathogen_Parasite	-10.40602114	0.00472038	0.698940246	0.000271213
	Parasite	-8.956513298	0.00038296	0.931527817	2.65857E-06
**MULTI_LIFESTYLE**	**Ectomycorrhizal-Undefined Saprotroph-Wood Saprotroph**	**-60.82121694**	**0.544875905**	**1.16919E-05**	**0.041427655**
	**multi-lifestyle**	**-7.606823761**	**-0.003691427**	**0.004429493**	**0.000336316**
	**Ectomycorrhizal-Fungal Parasite-Plant Saprotroph-Wood Saprotroph**	**-9.225987811**	**0.023580088**	**0.034213582**	**0.011210152**
	**Saprotroph_Ectomycorrhizal**	**-8.580824716**	**0.011413141**	**0.035823395**	**0.000526892**
	Pathogen_Saprotroph	-8.702076771	-0.002692807	0.157672857	5.50611E-05
	Parasite_Saprotroph	-8.392978504	-0.00452857	0.234019246	0.000204264
	Epiphyte-Leaf Saprotroph-Lichen Parasite-Lichenized-Plant Pathogen-Wood Saprotroph	-12.17060197	0.017861426	0.360963705	0.002068227
	Ectomycorrhizal_multi-lifestyle	-8.348265068	-0.002487667	0.626283158	1.19277E-05
**MYCORRHIZAL**	**Arbuscular_Mycorrhizal**	**-10.13194202**	**-0.025318155**	**0.000101068**	**0.005387098**
	Ectomycorrhizal	-7.590459839	0.001670372	0.108956507	2.00437E-05
	Orchid Mycorrhizal	-10.35730421	-0.01240599	0.142209808	0.001519002
	Mycorrhizal	-8.426148647	0.002810841	0.251862958	6.90074E-05
**SAPROTROPH**	Plant Saprotroph	-9.549150085	-0.016045731	0.530542732	0.000728374
	**Saprotroph**	**-8.697500686**	**-0.003681541**	**0.001060328**	**9.9928E-05**
	Leaf Saprotroph	-9.237159293	-0.03027083	0.089983212	0.012777341
	Soil Saprotroph	-8.790982741	0.003147962	0.208817998	9.33209E-05
**SYMBIOTIC**	Lichenized	-12.40850137	0.002433867	0.834236537	2.31951E-05
	Endophyte	-9.478079554	-0.000642862	0.845586559	7.50601E-06
**UNDEFINED**	**Undefined**	**-9.50058328**	**-0.003119321**	**0.001134873**	**4.89368E-05**

Table S10. GLM model coefficients for fungal guilds in relation to soil depth. For Guild 2, in bold fungal guilds responding to soil depth according to according to ANOVA ($p < 0.05$) of the GLM.

GUILD 1	GUILD 2	INTERCEPT	SLOPE	PR(>CHISQ)	R_VALUE
**PATHOGENS/PARASITES**	**Animal Pathogen**	**-6.155081825**	**-0.095479542**	**7.61829E-11**	**0.021094176**
	**Pathogen_Parasite**	**7.1336589**	**-1.617522553**	**7.13596E-09**	**0.025414419**
	**Plant Pathogen**	**-9.104729077**	**-0.061316388**	**0.00012329**	**0.001811655**
	Parasite	-8.388443104	-0.02872691	0.1542126	0.000979154
	Pathogen	-11.09153831	0.037275727	0.258485132	0.001241173
**MULTI_LIFESTYLE**	**Pathogen_Saprotroph**	**-8.048223742**	**-0.042843353**	**4.29237E-06**	**0.000799157**
	**Saprotroph_Ectomycorrhizal**	**-9.650858192**	**0.079039514**	**0.000600185**	**0.001680866**
	**multi-lifestyle**	**-7.48416705**	**-0.01619913**	**0.006511963**	**0.000340736**
	**Parasite_Saprotroph**	**-7.951377267**	**-0.035898957**	**0.043267296**	**0.000644664**
	Epiphyte-Leaf Saprotroph-Lichen Parasite-Lichenized-Plant Pathogen-Wood Saprotroph	-13.22763601	0.097120284	0.235203516	0.002202767
	Ectomycorrhizal-Fungal Parasite-Plant Saprotroph-Wood Saprotroph	-6.576235542	-0.056177554	0.25131575	0.002979663
	Ectomycorrhizal-Undefined Saprotroph-Wood Saprotroph	-6.231747953	-0.074688484	0.334933821	0.004864977
	Ectomycorrhizal_multi-lifestyle	-8.560851626	0.004011136	0.858244028	1.41585E-06
**MYCORRHIZAL**	**Orchid Mycorrhizal**	**-9.363702131**	**-0.091491266**	**0.036240145**	**0.003207986**
	Ectomycorrhizal	-7.668197432	0.008432225	0.071053614	2.3054E-05
	Arbuscular_Mycorrhizal	-11.81822563	0.03417602	0.179472645	0.000394003
	Mycorrhizal	-8.364079797	0.004816005	0.665682131	1.23196E-05
**SAPROTROPH**	Plant Saprotroph	-10.25144093	-1.04572E-15	1	0
	**Soil Saprotroph**	**-7.896259141**	**-0.038208585**	**0.00042864**	**0.000802776**
	Saprotroph	-8.764202425	-0.006334415	0.216500171	1.56939E-05
	Leaf Saprotroph	-11.32101709	0.047050209	0.484813575	0.001145878
**SYMBIOTIC**	Lichenized	-12.61290742	0.016617662	0.743754334	8.32453E-05
	Endophyte	-9.561033665	0.002393841	0.872187177	4.31054E-06
**UNDEFINED**	**Undefined**	**-9.488225069**	**-0.00894877**	**0.040082354**	**2.14173E-05**

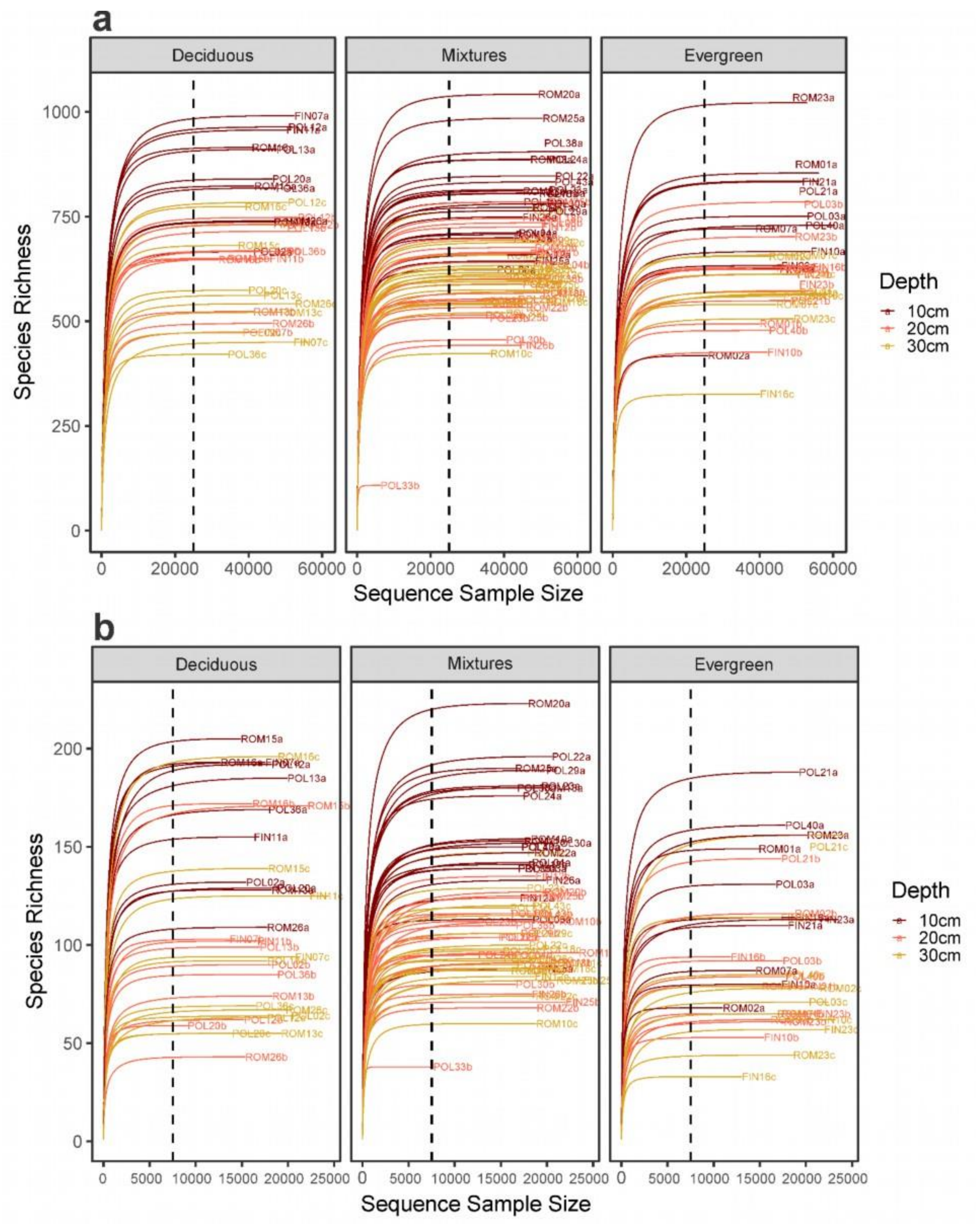

Figure S1. Rarefaction curves of bacterial (a) and fungal (b) of 132 soil samples. Soil samples from the three forest types represent 16,358 bacterial ASVs and 4,635 fungal ASVs.

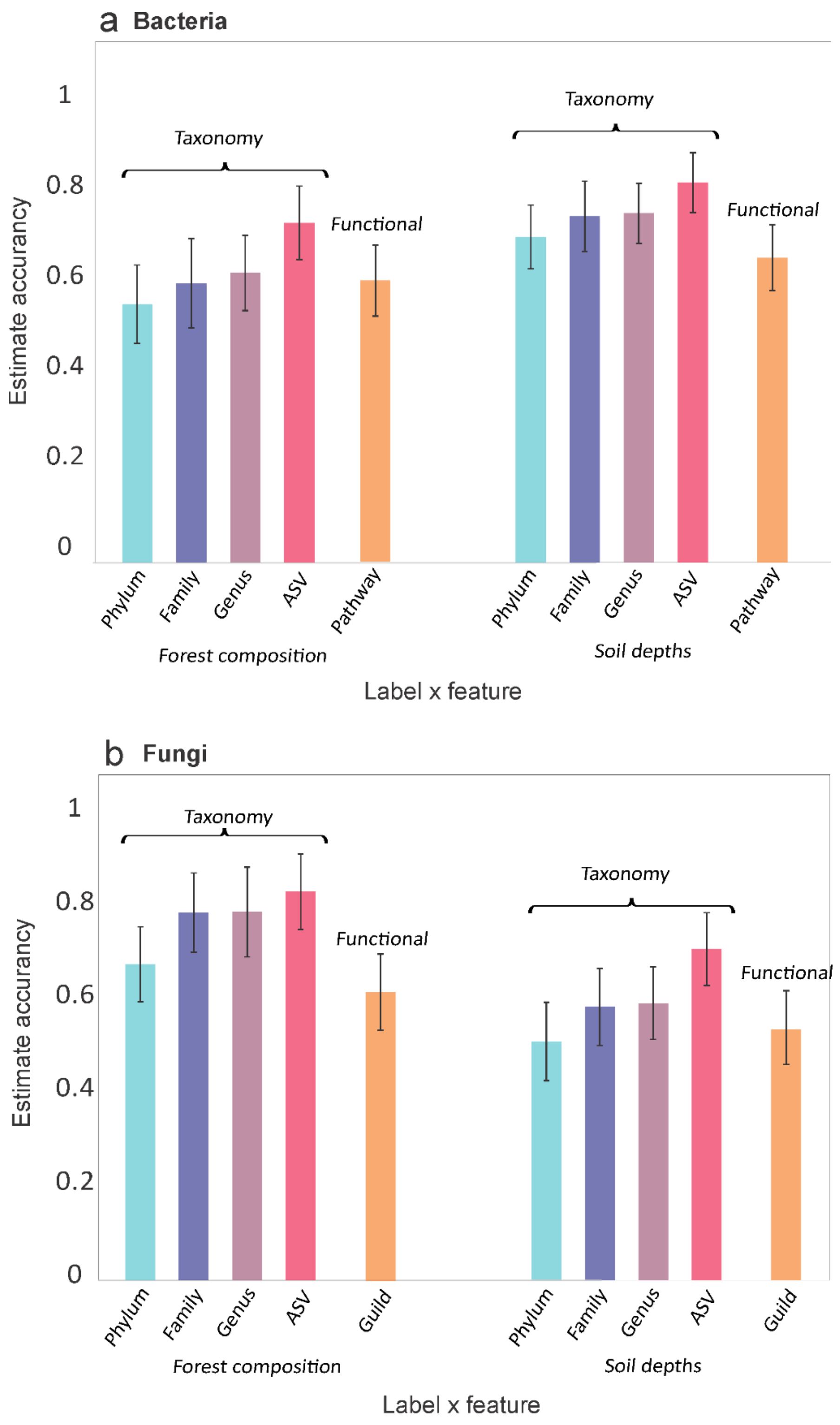

Figure S2. Estimated accuracy of random forest model for bacteria (a) and fungi (b). Bars represent average of 100 runs for each combination. Error bars represent standard error (N = 100).

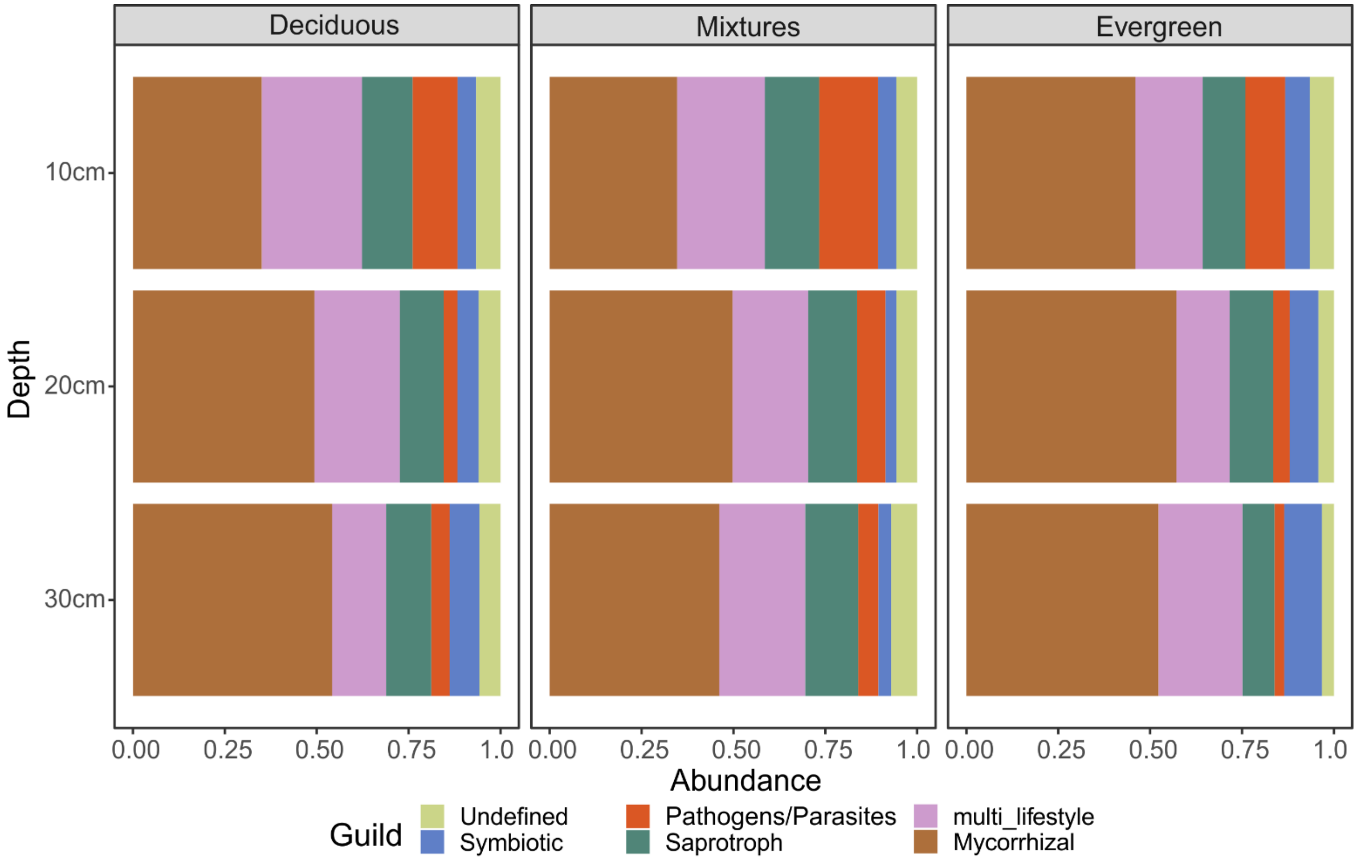

Figure S3. Relative abundances by guilds in relation to forest composition and soil depth.
